# A comparative analysis of Marburg virus-infected bat and human models from public high-throughput sequencing data

**DOI:** 10.7150/ijms.100696

**Published:** 2025-01-01

**Authors:** Do Thi Minh Xuan, I-Jeng Yeh, Hsin-Liang Liu, Che-Yu Su, Ching-Chung Ko, Hoang Dang Khoa Ta, Jia-Zhen Jiang, Zhengda Sun, Hung-Yun Lin, Chih-Yang Wang, Meng-Chi Yen

**Affiliations:** 1 Faculty of Pharmacy, Van Lang University, 69/68 Dang Thuy Tram Street, Ward 13, Binh Thanh District, Ho Chi Minh City 70000, Vietnam.; 2 Department of Emergency Medicine, Kaohsiung Medical University Hospital, Kaohsiung Medical University, Kaohsiung 80708, Taiwan.; 3 Graduate Institute of Clinical Medicine, College of Medicine, Kaohsiung Medical University, Kaohsiung 80708, Taiwan.; 4 Department of Medical Imaging, Chi-Mei Medical Center, Tainan, Taiwan.; 5 Department of Health and Nutrition, Chia Nan University of Pharmacy and Science, Tainan, Taiwan.; 6 School of Medicine, College of Medicine, National Sun Yat-Sen University, Kaohsiung, Taiwan.; 7 Ph.D. Program for Cancer Molecular Biology and Drug Discovery, College of Medical Science, Taipei Medical University, Taipei 11031, Taiwan.; 8 Emergency Department, Huashan Hospital North, Fudan University, Shanghai 201508, People's Republic of China.; 9 Kaiser Permanente, Northern California Regional Laboratories, The Permanente Medical Group, 1725 Eastshore Hwy, Berkeley, CA 94710, USA.; 10 Graduate Institute of Cancer Biology and Drug Discovery, College of Medical Science and Technology, Taipei Medical University, Taipei 11031, Taiwan.; 11 Cancer Center, Wan Fang Hospital, Taipei Medical University, Taipei 11031, Taiwan.; 12 TMU Research Center of Cancer Translational Medicine, Taipei Medical University, Taipei 11031, Taiwan.; 13 Traditional Herbal Medicine Research Center of Taipei Medical University Hospital, Taipei Medical University, Taipei 11031, Taiwan.; 14 Pharmaceutical Research Institute, Albany College of Pharmacy and Health Sciences, Rensselaer, NY 12144, USA.

**Keywords:** Marburg virus (MARV), *Rousettus aegyptiacus*, *Homo sapiens*, zoonotic disease, bioinformatics

## Abstract

Marburg virus (MARV) disease (MVD) is an uncommon yet serious viral hemorrhagic fever that impacts humans and non-human primates. In humans, infection by the MARV is marked by rapid onset, high transmissibility, and elevated mortality rates, presenting considerable obstacles to the development of vaccines and treatments. Bats, particularly *Rousettus aegyptiacus*, are suspected to be natural hosts of MARV. Previous research reported asymptomatic MARV infection in bats, in stark contrast to the severe responses observed in humans and other primates. Recent MARV outbreaks highlight significant public health concerns, underscoring the need for gene expression studies during MARV progression. To investigate this, we employed two models from the Gene Expression Omnibus, including kidney cells from *Rousettus aegyptiacus* and primary proximal tubular cells from *Homo sapiens*. These models were chosen to identify changes in gene expression profiles and to examine co-regulated genes and pathways involved in MARV disease progression. Our analysis of differentially expressed genes (DEGs) revealed that these genes are mainly associated with pathways related to the complement system, innate immune response via interferons (IFNs), Wnt/β-catenin signaling, and Hedgehog signaling, which played crucial roles in MARV infection across both models. Furthermore, we also identified several potential compounds that may be useful against MARV infection. These findings offer valuable insights into the mechanisms underlying MARV's pathophysiology and suggest potential strategies for preventing transmission, managing post-infection effects, and developing future vaccines.

## Introduction

Marburg virus (MARV) was first identified in 1967 and is classified as a negative-sense RNA virus from the Filoviridae family. It shares a close genetic relationship with Ebola virus, both known to cause severe hemorrhagic fever in humans and non-human primates, generally known as MARV disease (MVD) and Ebola virus disease (EVD), respectively. The MARV genus consists of two variants, MARV and Ravn virus (RAVV), whose overall genetic sequences exhibit a genetic divergence of approximately 20%. However, both variants lead to a clinically indistinguishable form of MVD characterized by multiple hemorrhagic manifestations with a case fatality rate that can range up to 80%-90% as recorded in previous outbreaks^1^. In the last two decades, subsequent outbreaks of MVD were found to be sporadic and endemic to sub-Saharan Africa, characterized by the continuous introduction of both MARV and RAVV from fruit bats, especially the cave-dwelling Egyptian fruit bat (*Rousettus aegyptiacus*), which was identified as a wildlife reservoir^2^. African green monkeys are the primate spillover hosts whose infected tissues were the cause of seven fatalities in the first outbreaks that were simultaneously documented in Marburg, Germany, and Belgrade, Yugoslavia (now Serbia). As of 30 September 2024, in Disease Outbreak News reported by the WHO (https://www.who.int/emergencies/disease-outbreak-news/item/2024-DON537), during the most recent and largest outbreak in new geographical areas such as Rwanda, Equatorial Guinea, and Tanzania, MAVR was documented to spread through human-to-human transmission via direct exposure to blood, bodily fluids, and secretions of sick patients. Indirect transmission occurs through close contact with materials or surfaces contaminated with the virus from infected humans and animals^3^-^5^. This has led to increased public health concerns and heightened media attention on MVD, despite the fact that MARVs have historically received less attention compared to the Ebola virus^6^, ^7^.

As of now, documented information regarding the fundamental processes of viral transmission and disease pathways remains limited, thus presenting challengs to research into specific antiviral treatments and vaccines for MVD. Therefore, comprehending the dynamics of MARV transmission between its natural host reservoirs and humans has become crucial for outbreak prevention and control. Although accurate animal models that mirror the human pathogenesis are indispensable for drug development, the US FDA's animal efficacy rule provides a framework for utilizing data from such models when conducting human trials is not feasible^8^. Notably, differences in a host's responses to viral infection among infected animals have been recorded, suggesting that the relevant pathogenesis and immunity following MARV replication may vary among reservoirs. In the case of MARV, previous research reported the asymptomatic infection of this virus in natural bat reservoirs, which was completely opposed to the severe immune responses and inflammatory gene dysregulation recorded in their spillover hosts including humans and non-human primates following viral infection^9^-^11^. This difference is believed to primarily be caused by the reverse regulation pattern of interferon (IFN) responses and proinflammatory disease mediators in these two hosts, resulting in either the induction or suppression of antiviral responses^12^. However, to date, no studies have investigated similar patterns of viral infection in bats and humans during the incubation period, which could help characterize the entry and dissemination of MARV in diferent hosts as well as subsequent disease progression.

In addition to lymphoid tissues and the liver, which are well-known targets of filovirus infection, previous laboratory findings showed a link between renal dysfunction and patients in the late stages of MVD, as MARV virions were detected in renal tissues and MARV antigens were present in proximal tubular cells^13^, ^14^. Supporting these findings, Martini *et al.* in 1971 and Smith *et al.* in 1982 respectively reported the presence of live virus isolated from kidney tissues and urine^15^, ^16^. In 2018, Arnold *et al.* conducted a transcriptomic analysis of two models: an *Egyptian rousette* bat (ERB) kidney-derived cell line (RoNi 7.1) infected with either a wild-type MARV or a recombinant mutant MARV (VP35mut) compared to their respective mock-infected groups^17^. The major finding of that work was that immune suppression remained a critical feature of MARV infection in the *in vitro* ERB model via upregulation of antiviral genes. Another study conducted by Koch *et al.* in 2023 applied transcriptome analyses at multiple time points in primary human proximal tubular cells and revealed that the *in vitro* productive replication of MARV was correlated with elevated levels of IFN-related factors and cytokines in the early phase of viral infection. This strong inflammatory and antiviral response was linked to kidney injury^18^. Therefore, investigating how reverse regulation patterns of related genes and pathways in the two MARV-infected *in vitro* models of humans and bats are related to antiviral responses would be a highly valuable research study.

**Figure 1:**
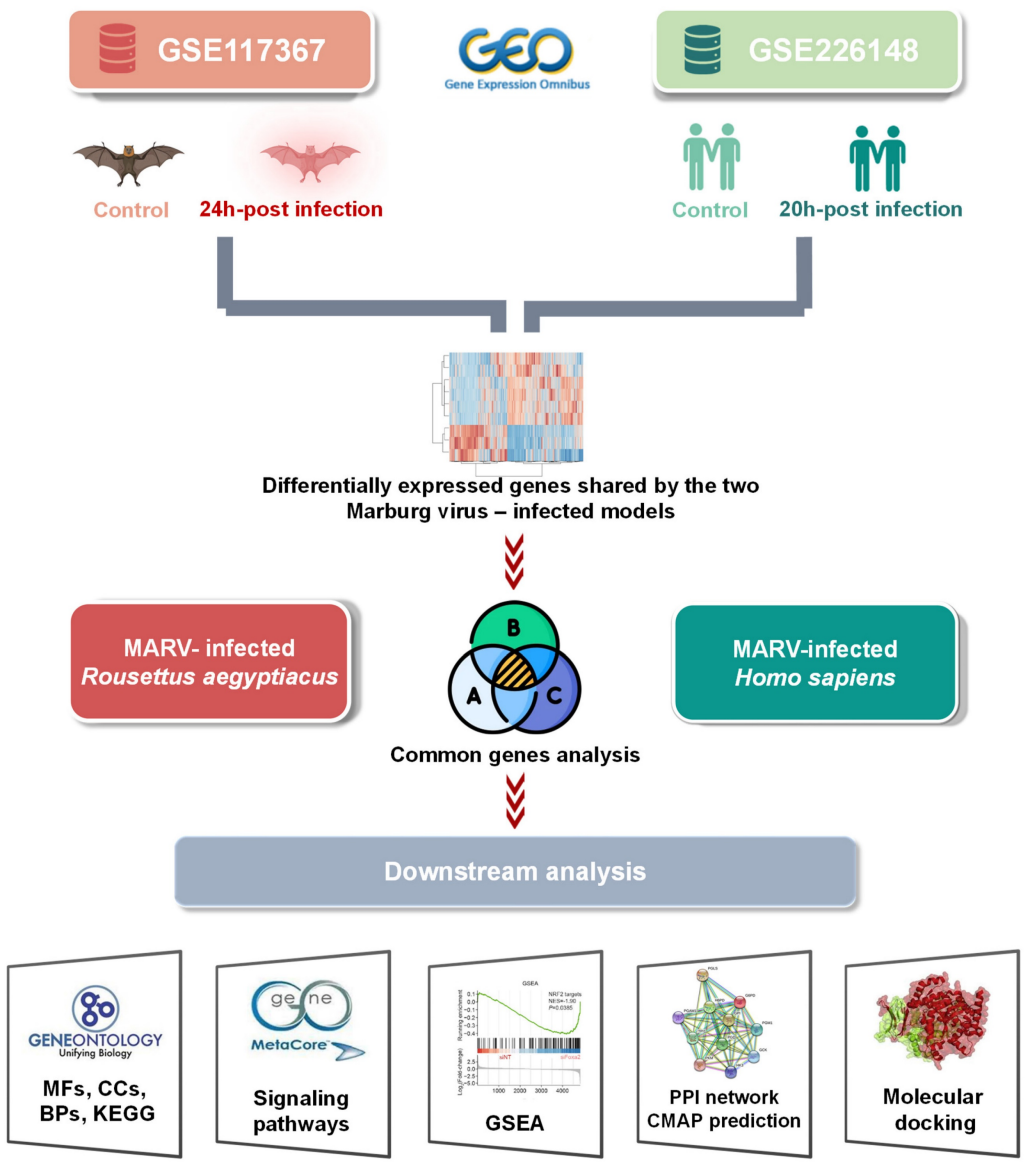
** Graphical abstract of the study design.** Data were obtained from the Marburg virus (MARV)-infected RoNi/7.1 cell line, derived from *Rousettus aegyptiacus*, and from MARV-infected primary proximal tubular cells from *Homo sapiens* in the Gene Expression Omnibus (GEO) database. By crossing fold change > 2.0 upregulated genes (MARV-infected groups vs. a mock-infected control groups) in each MARV-infected model using a Venn diagram analysis, common genes were projected to pathway analyses and functional interpretations using bioinformatics approaches.

High-throughput technologies have become essential in the systematic investigation of expression differences of thousands of genes across various biological and genomic systems^19^-^25^. In recent years, the integration of multiple high-throughput databases and analytical tools has paved the way for big data approaches in biomedical research^26^-^28^, offering new insights and enhancing the precision of data-driven discoveries^29^-^34^. In this work, we aimed to investigate key similarities and distinctions in gene expression patterns of two *in vitro* MARV-infected kidney models—one from humans and one from bats—using sequencing data from previous studies. This includes data from the MARV-infected immortalized RoNi/7.1 kidney cell line, derived from the fruit bat *R. aegyptiacus*, which was identified as a reservoir host of MARV (GSE117367), and from MARV-infected *H. sapiens* primary proximal tubular cells, compared to a mock-infected group (GSE226148). Pathways regulated by top exclusive and shared differentially expressed genes (DEGs) between these two MARV-infected cell line models may provide a more-insightful understanding of key factors that contribute to the disparate outcomes between reservoir hosts and spillover hosts during MARV progression (Figure 1), guiding the development of effective antiviral therapies in the near future.

## Materials and Methods

### Data acquisition and processing methods

In the current study, our objective was to investigate the effects of MARV infection on gene expressions from two distinct MARV-infected subjects: the RoNi/7.1 immortalized kidney cell line derived from a MARV-infected Egyptian fruit bat (R. aegyptiacus) and primary proximal tubular cells derived from H. sapiens. Relevant transcriptomic datasets were sourced from the NCBI's Gene Expression Omnibus (GEO; www.ncbi.nlm.nih.gov/geo). Transcriptomic profiles of the 24-h MARV-infected R. aegyptiacus RoNi/7.1 cell line and their mock-infected counterparts were extracted from the GSE117367 dataset^18^, while similar information for 20-h MARV-infected H. sapiens proximal tubular cells was retrieved from the GSE226148 dataset^17^. Two NCBI-generated RNA-sequencing (RNA-Seq) count datasets underwent processing and normalization using DESeq2^35^. Comparisons with mock-infected groups were conducted to identify DEGs in each model^36^-^38^. Gene groups with significant annotations were selected using a cutoff of 0.05 (p < 0.05). Clustering based on expression profiles was performed using Kallisto following guidance of Jayaprakash *et al.*^39^, ^40^. The most substantial fold changes, with absolute values exceeding 1.5, were then mapped onto the Uniform Manifold Approximation and Projection (UMAP)^41^, and subjected to gene ontology (GO) and Kyoto Encyclopedia of Genes and Genomes (KEGG) analyses^42^, ^43^., all integrated using Omics Playground v.3.4.1^44^.

### MetaCore pathway analysis

An additional functional enrichment analysis was conducted using MetaCore™ (GeneGo, St. Joseph, MN, USA) to comprehensively analyze biological pathways associated with the distinctive and common DEGs (with fold changes of > 1.5) between the two models. The significant pathways in each case were ranked in descending order of -log10(p value) as we previous described^45^, ^46^..

### Gene set enrichment analysis (GSEA)

A GSEA was performed to compare enriched gene sets in the two MARV-infected datasets using the Python GSEApy package^47^. The cutoff for significant enrichment was set to a false discovery rate (FDR) of < 0.25^48^, ^49^.

### Protein-protein interaction (PPI) network construction using STRING and Cytoscape

The STRING database, vers. 12.0 (accessible at https://string-db.org/), functions as both a search engine and a resource for protein-related information. It offers an extensive collection of proteins and established interaction data used to analyze DEGs in this study. Additionally, to visualize and interpret the complex interaction networks among the identified proteins, Cytoscape software was utilized to construct a detailed PPI network, and the k-means clustering algorithm was employed to further categorize proteins into distinct clusters based on their interaction patterns, facilitating a more-granular analysis of their roles and relationships within the biological processes under study.

### Constructing a connectivity map (CMap) of potential therapeutic chemical compounds

CMap employs a systematic methodology to analyze changes in gene expressions to uncover disease interactions and match those with compounds listed in the LINCS L1000 database^50^. Common DEGs shared between the two MARV-infected models, compared to their mock-infected groups, were projected to the CMap platform to construct a connectivity map of potential compounds that may induce or reverse the biological effects or counteract gene expression changes observed in the query. Normalized connectivity scores indicate both the direction and intensity of the relationship between a query (such as a gene expression signature) and the perturbagen profiles (such as drugs or small molecules) found in the LINCS database. A positive score suggests that the gene expression signature induced by the perturbagen closely resembles the query signature, whereas a negative score indicates that the gene expression signature induced by the perturbagen is the opposite of the query signature.

### Statistical analysis

Statistical analyses in this study were conducted using R vers. 4.1.3. The Wilcoxon test, a non-parametric method ideal for comparing two independent samples, was used to evaluate differences between various phenotypic groups. To correct for multiple comparisons, *p* values obtained from the Wilcoxon test were adjusted using the FDR method. A *p* value of < 0.05 was regarded as statistically significant, indicating meaningful differences between the groups analyzed.

## Results

### Clustering and GO analytical results of DEGs from the two MARV-infected models

**Figure 2:**
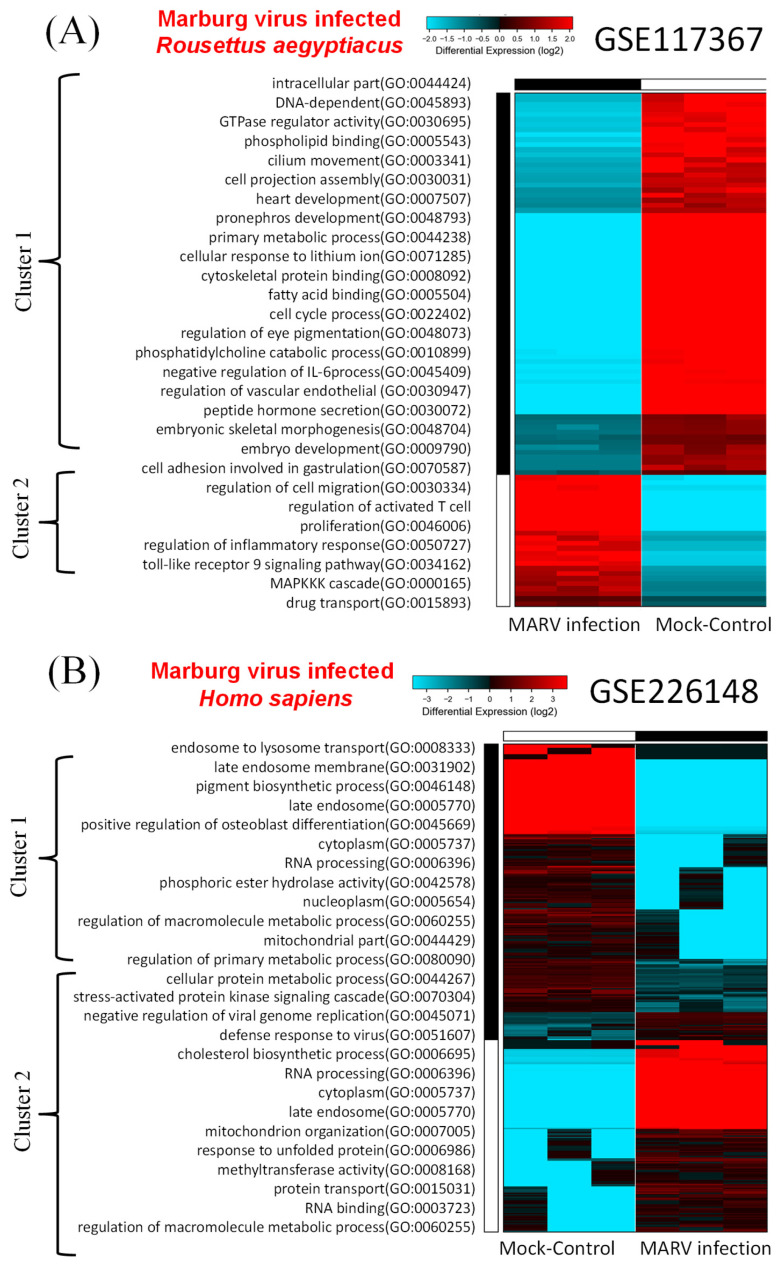
**Hierarchical clustered heatmaps of gene ontology (GO) enrichment. A** GO enrichment analysis was performed on sets of differentially expressed genes (DEGs) within the two Marburg virus (MARV)-infected models compared to corresponding mock-infected groups, including (A) a MARV-infected bat-derived cell line at 24 h post-infection, and (B) a MARV-infected human-derived cell line at 20 h post-infection.

In the model involving the MARV-infected bat-derived RoNi/7.1 cell line, we conducted a comparative analysis of DEGs using transcriptomic data from the GSE117367 dataset. Figure 2A illustrates the results of the GO analysis associated with MARV infection. Cluster 2 contained the most significantly upregulated genes and pathways in the MARV-infected group, while cluster 1 exhibited the opposite trend. These pathways within cluster 1 were linked to intracellular components and cell development, such as intracellular parts (GO:0044424), DNA-dependent (GO:0045893), GTPase regulator activity (GO:0030695), phospholipid binding (GO:0005543), cilium movement (GO:0003341), cell projection assembly (GO:0030031), heart development (GO:0007507), pronephros development (GO:0048793), primary metabolic processes (GO:0044238), cellular response to lithium ions (GO:0071285), cytoskeletal protein binding (GO:0008092), fatty acid binding (GO:0005504), cell cycle processes (GO:0022402), regulation of eye pigmentation (GO:0048073), phosphatidylcholine catabolic processes (GO:0010899), negative regulation of interleukin (IL)-6 processes (GO:0045409), regulation of the vascular endothelium (GO:0030947), peptide hormone secretion (GO:0030072), embryonic skeletal morphogenesis (GO:0048704), embryo development (GO:0009790), cell adhesion involved in gastrulation (GO:0070587), and regulation of cell migration (GO:0030334). In contrast, the GO analysis revealed that cluster 2 was associated with inflammatory responses, chemokine activities, and immune system processes (Figure 2A). These included regulation of cell migration (GO:0030334), regulation of activated T-cell proliferation (GO:0046006), regulation of inflammatory responses (GO:0050727), toll-like receptor 9 signaling pathway (GO:0034162), mitogen-activate protein kinase (MAPK) kinase kinase (MAPKKK) cascade (GO:0000165), and drug transport (GO:0015893).

For the MARV-infected human-derived cell line model, transcriptomic raw data were obtained from the GSE226148 dataset. In this case, pathways within cluster 2 were strongly associated with MARV infection, while cluster 1 displayed an opposing trend (Figure 2B). The GO analysis indicated that pathways within cluster 1 were related to endosome function and metabolism; these included processes such as endosome to endosome to lysosome transport (GO:0008333), late endosome membranes (GO:0031902), pigment biosynthetic processes (GO:0046148), late endosomes (GO:0005770), positive regulation of osteoblast differentiation (GO:0045669), cytoplasm (GO:0005737), RNA processing (GO:0006396), phosphoric ester hydrolase activity (GO:0042578), nucleoplasm (GO:0005654), regulation of macromolecule metabolic processes (GO:0060255), mitochondrial parts (GO:0044429), regulation of primary metabolic processes (GO:0080090), cellular protein metabolic processes (GO:0044267), and cellular protein metabolic processes (GO:0044267). On the other hand, pathways within cluster 2 were linked to regulation of viral infections and stress-related signaling, such as stress-activated protein kinase signaling cascade (GO:0070304), negative regulation of viral genome replication (GO:0045071), defense response to viruses (GO:0051607), cholesterol biosynthetic processes (GO:00066695), RNA processing (GO:0006396), cytoplasm (GO:0005737), late endosomes (GO:0005770), mitochondrion organization (GO:0007005), response to unfolded proteins (GO:0006986), methyltransferase activity (GO:0008168), protein transport (GO:0015031), RNA binding (GO:0003723), and regulation of macromolecule metabolic processes (GO:0060255).

### UMAP clustering of gene sets and relative GO annotations of unique and shared highly expressed genes in the two MARV-infected models

**Figure 3:**
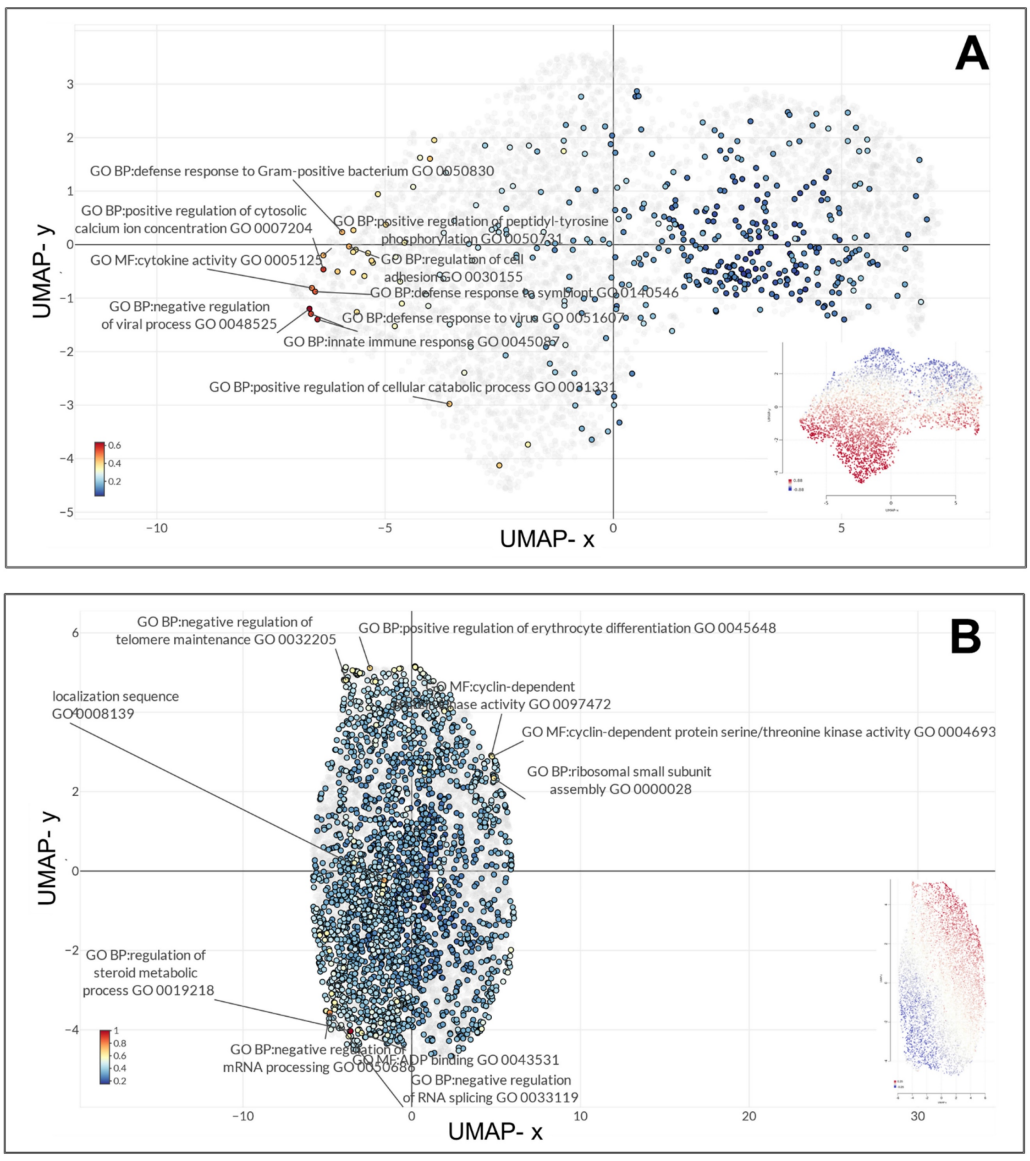
** UMAP clustering of top enriched gene ontology (GO) terms represented for the top distinct diferentially expressed genes in the two Marburg virus (MARV)-infected models.** The top 10 most enriched GO terms across biological process, molecular function, and cellular component categories in (A) a MARV-infected bat-derived cell line at 24 h post-infection, and (B) a MARV-infected human-derived cell line at 20 h post-infection.

Gene expression profiles of the MARV-infected bat-derived RoNi/7.1 immortalized kidney cell line (GSE117367) and MARV-infected human-derived primary proximal tubular cell line (GSE226148) were analyzed using the GO platform integrated with Omics Playground for biological process, molecular function, and cellular component annotations specific to MARV infection conditions. In the former model, these genes primarily functioned in endothelial cell development (GO_0001885), tumor necrosis factor (TNF) receptor superfamily binding (GO_0032813), regulation of c-Jun N-terminal kinase (JUNK) activity (GO_0043506), negative regulation of protein localization to membranes (GO_1905476), determination of bilateral symmetry (GO_0009855), RNA cap binding (GO_0000339), mitotic spindle assembly checkpoint signaling (GO_0007094), mitotic spindle checkpoint signaling (GO_0071174), spindle assembly checkpoint signaling (GO_0071173), and axoneme assembly (GO_0035082) (Figure 3A). In the latter model, the most significant biological terms involved were negative regulation of viral processes (GO_0048525), innate immune responses (GO_0045087), positive regulation of cytosolic calcium ion concentrations (GO_0007204), defense responses to viruses (GO_0051607), defense responses to symbionts (GO_0140546), cytokine activity (GO_0005125), regulation of cell adhesion (GO_0030155), defense responses to gram-positive bacterium (GO_0050830), positive regulation of the MAPK cascade (GO_0043410), and negative regulation of cytokine production (GO_0001818) (Figure 3B). Major characteristics of BP annotations for each MARV-infected model are summarized in Figure 5A with details in Supplementary Table A2. The annotations are organized in descending order of -log(*p* values), highlighting the most significantly enriched processes associated with the DEGs in each model.

### Pathway analysis results and pathway enrichment analysis results of distinct and common DEGs shared between the two MARV-infected models

**Figure 4:**
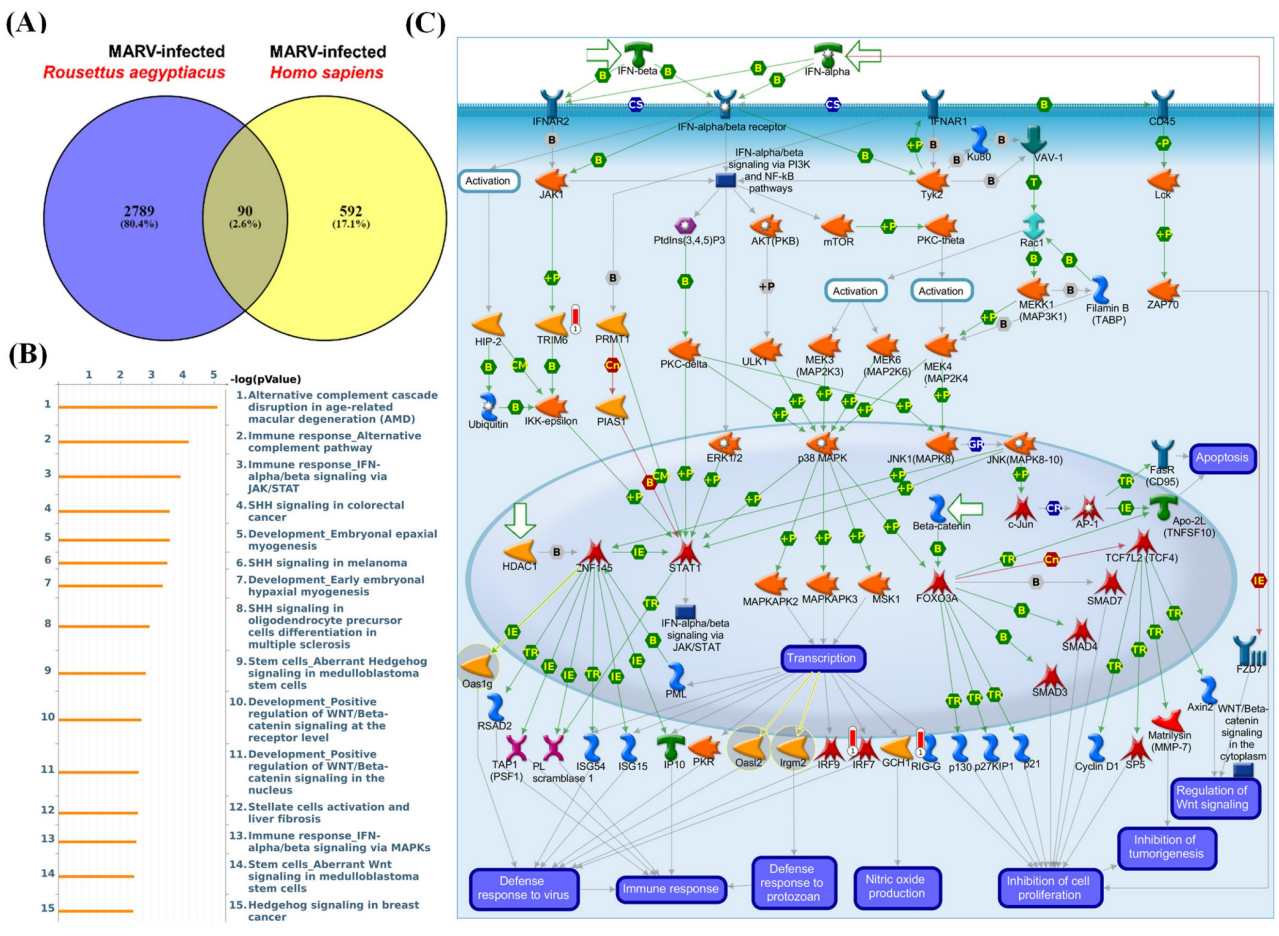
** Comparison of differentially expressed gene (DEG)-enriched signaling pathways in Marburg virus (MARV)-infected models.** (A) The Venn diagram illustrates the number of distinct and common DEGs (with fold change of > 1.2) when comparing MARV-infected groups to their respective mock-infected control groups within the GSE117367 and GSE226148 datasets. The overlapping region represents genes shared between the two datasets, while the non-overlapping areas indicate genes unique to each dataset. (B) List of the top 15 DEG-enriched signaling pathways regulated by the common DEGs, generated by MetaCore, and sorted in descending order of log(*p* values). (C) Visualization of the global signal transduction pathway network generated by MetaCore confirmed that the “Immune response_IFN-alpha/beta signaling via JAK/STAT” pathway was highly enriched in both MARV-infected models.

Since the MARV is a zoonotic disease observed in the context of emerging zoonotic infectious diseases, the dynamics underlying the shifting epidemiology can be attributed to multiple factors. These include variations related to pathogen evolution, characteristics of the zoonotic reservoir, and nuances of the human-animal interface. In order to explore the co-regulation of genes for both *in vitro* MARV-infected bat and human models, we employed Venn diagrams to identify common DEGs shared between the MARV-infected human model from the GSE226148 dataset and the MARV-infected *R. aegyptiacus* model from the GSE117367 dataset, specifically retrieving overlapping DEGs with a fold change of > 1.5 (Figure 4A, Supplementary Table A3). Altogether, we identified 463 common DEGs shared between these two MARV-infected models, and then subsequently subjected these genes to a MetaCore analysis, which revealed several standard maps related to MARV infection, including pathways such as: “Alternative complement cascade disruption in age-related macular degeneration (AMD)”, “Immune response_Alternative complement pathway”, “Immune response_IFN-alpha/beta signaling via JAK/STAT”, “SHH signaling in colorectal cancer”, “Development_Embryonal epaxial myogenesis”, “SHH signaling in melanoma”, “Development_Early embryonal hypaxial myogenesis”, “SHH signaling in oligodendrocyte precursor cells differentiation in multiple sclerosis”, “Stem cells_Aberrant Hedgehog signaling in medulloblastoma stem cells”, “Development_Positive regulation of WNT/Beta-catenin signaling at threceptor level”, “Development_Positive regulation of WNT/Beta-catenin signaling in the nucleus”, “Stellate cells activation and liver fibrosis”, “Immune response_IFN-alpha/beta signaling via MAPKs”, and “Stem cells Aberrant Wnt signaling in medulloblastoma stem cells. Hedgehog signaling in breast cancer”. Notably, signaling pathways related to complement signaling, IFN-alpha/beta response, WNT/Beta-catenin signaling, and Hedgehog (HH) signaling were highly enriched among the common DEGs between these two models (Figure 4C).

To gain a deeper understanding of the biological processes in each model, we conducted separate analyses of pathways related to MARV infection. In the case of the MARV-infected *R. aegyptiacus* model, 3212 DEGs with an absolute fold change of > 1.5 (MARV-infected groups vs. mock-infected control groups) were subjected to a MetaCore analysis which revealed significant maps such as: “Role of ER stress in obesity and type 2 diabetes”, “DNA damage_p53 activation by DNA damage”, “Apoptosis and survival_p53 and p73-dependent apoptosis”, “SCAP/SREBP Transcriptional Control of Cholesterol and FA Biosynthesis”, “Development_Positive regulation of WNT/Beta-catenin signaling in the cytoplasm”, “Development_Negative regulation of WNT/Beta-catenin”, “Transcription_Epigenetic regulation of gene expression”, “Regulation of metabolism_Negative regulation of insulin signaling”, “Signal transduction_mTORC1 downstream signaling”, “Signal transduction_ESR1 (membrane) and ESR2 (membrane) signaling”, “Signal transduction_mTORC2 downstream signaling”, “Development_Negative regulation of WNT/Beta-catenin signaling in the nucleus”, “Chemotaxis_Lysophosphatidic acid signaling via GPCRs”, “G-protein signaling_CDC42 activation,” and “Autophagy_Autophagy” (Supplementary Figure 1A-B, Supplementary Table A4). For the MARV-infected *H. sapiens* model, 682 DEGs with an absolute fold change of > 1.5 (MARV-infected groups vs. mock-infected control groups) were subjected to a MetaCore analysis which revealed significant maps such as “Immune response_IFN-alpha/beta signaling via JAK/STAT”, “COVID-19: Regulation of antiviral response by SARS-CoV-2”, “Immune response_IFN-alpha/beta signaling via MAPKs”, “Cigarette smoke-mediated regulation of NRF2-antioxidant pathway in airway epithelial cells”, “Attenuation of IFN type I signaling in melanoma cells”, “Inhibition of Ephrin receptors in colorectal cancer”, “Immune response_IFN-gamma signaling via MAPKs”, “Immune response_Antiviral actions of Interferons”, “Immune response_Antimicrobial actions of IFN-gamma”, “Zika virus infection mechanism” “Immune response_Induction of apoptosis and inhibition of proliferation mediated by IFN-gamma”, “WNT signaling in proliferative-type melanoma cells”, “Glomerular injury in Lupus Nephritis”, “Transcription_HIF-1 targets”, and “Role of Apo-2L(TNFSF10) in Prostate Cancer cell apoptosis gamma” (Supplementary Figure 2A, B, Supplementary Table A5).

**Figure 5:**
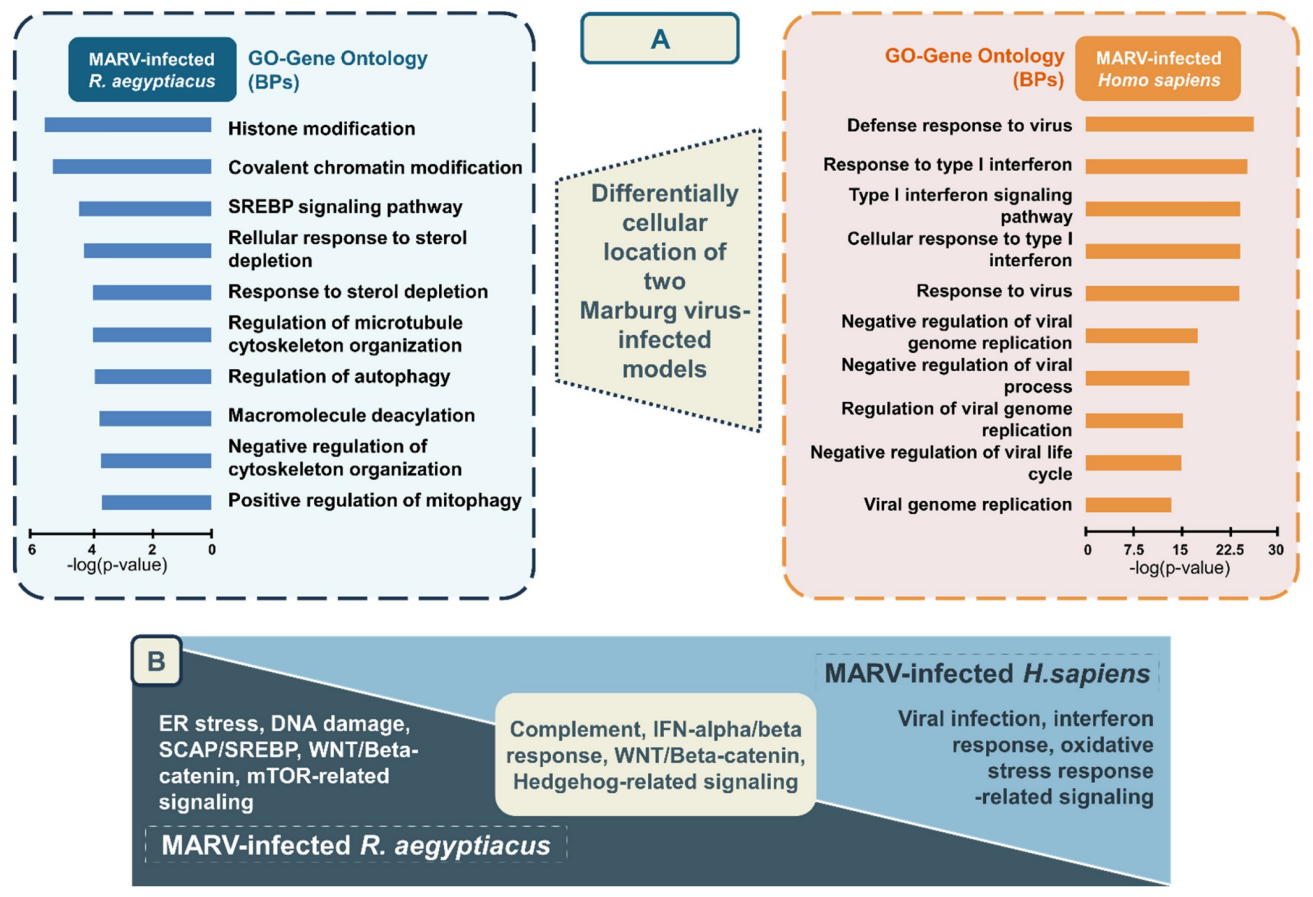
** Summary visualization of common and distinct biological process (BP) gene ontology (GO) terms and pathway analysis associated with the top differentially expressed genes (DEGs) in two Marburg virus (MARV)-infected *in vitro* models.** The top enriched GO biological process terms in (A) the MARV-infected *Rousettus aegyptiacus in vitro* model, and (B) the MARV-infected *Homo sapiens in vitro* model. (C) Distinctive and common pathways regulated by the top DEGs in the two MARV-infected models at a cutoff log2 fold change of > 1.5 and a *p* value of < 0.05.

In summary, pathways related to complement signaling, IFN-alpha/beta response, WNT/Beta-catenin signaling, and HH signaling were identified as enriched pathways regulated by common leading edge genes under MARV infection conditions in both species. Conversely, endoplasmic reticular (ER) stress, DNA damage, WNT/Beta-catenin, and sterol regulatory element-binding protein (SREBP) cleavage-activating protein (SCAP)/SREBP were significantly enriched signaling-related pathways in the MARV-infected *R. aegyptiacus*-derived RoNi/7.1 immortalized kidney cell line, whereas pathways related to viral infection response, IFN-alpha/beta signaling, and oxidative stress response were notably upregulated in the MARV-infected *H. sapiens* primary proximal tubular cell line (Figure 5B). In addition, pathways identified through the GSEA for each MARV-infected model were consistent with the above findings (Supplementary Figure 3).

### Construction of a PPI Network and potential inhibitory compounds from the connectivity map

**Figure 6:**
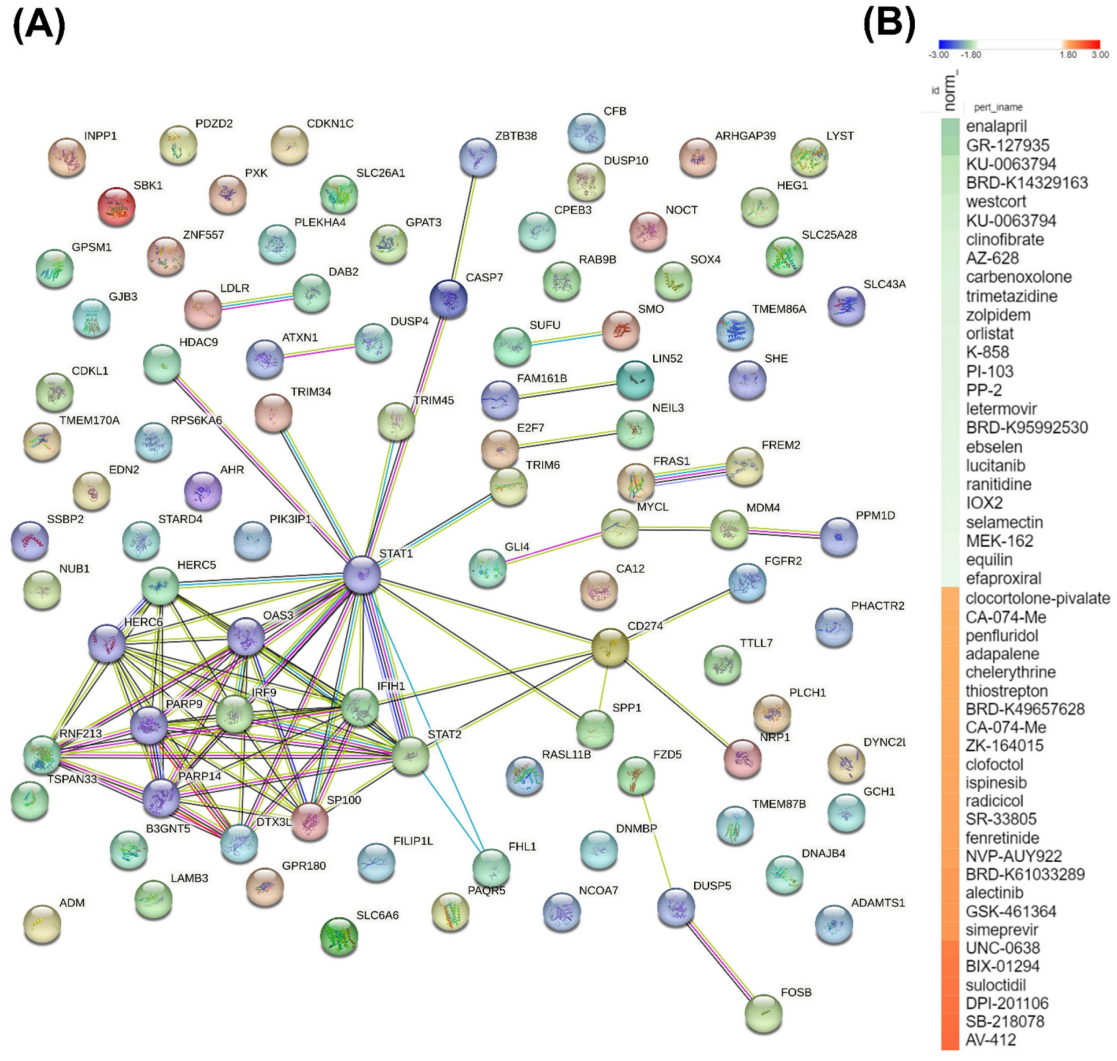
** A key protein-protein-interacting (PPI) network and list of potential compounds from the connectivity map (CMAP)-based analysis.** (A) The 90 differentially expressed genes (DEGs) shared between two MARV-infected groups, compared to the mock control group of both models, namely the Marburg virus (MARV)-infected *Rousettus aegyptiacus* (GSE117367) and MARV-infected *Homo sapiens* (GSE226148), were subjected to a functional protein association network analysis using the STRING database and the k-means clustering algorithm (*n*=10). (B) The CMap analysis presents a significant connectivity score when querying gene expression changes of 90 genes of interest against compounds from the LINCS L1000 database. Small-molecule compounds that caused similar gene expression signatures resulted in a positive correlation (red), while those causing opposing gene expression signatures resulted in a negative correlation (green). The top 50 potential compounds were sorted in descending order based on normalization of connectivity scores (norm_cs) at a cutoff false discovery rate (FDR)_q_nlog10 = 2.

To further investigate the significance of commonly shared DEGs between the two MARV-infected models, we used a Venn diagram to identify 90 genes that were differentially expressed in both MARV-infected groups (compared to mock-infected control groups) with a fold change of > 1.5. These 90 genes were then imported into the STRING platform to construct PPI networks, and we applied a k-means clustering algorithm with *n*=10, aiming to understand their potential roles in manifestations following a MARV infection (Figure 6A). Subsequently, we employed a CMap to identify compounds that could potentially mimic or reverse the 90 gene expression profiles in these two MARV-infected models. We screened over 1309 compounds and more than 7000 treatments with various dosages across different cell lines, selecting potential therapeutic agents based on an FDR q-value threshold of 2.0 (negative log10-transformed FDR q-values) and sorting by normalized connectivity scores (norm_cs). Compounds were considered promising if they demonstrated a negative norm_cs, as they may reverse the biological effects or counteract gene expression changes observed in the query. The top 25 compounds with the highest positive and negative connectivity scores were each respectively visualized in red and green in the CMap in the form of a heatmap (Figure 6B). In total, 117 perturbagens showed a positive correlation, while 98 perturbagens showed a negative correlation to the 90 queried genes (Supplementary Table A6, Supplementary Table A7). Among the 98 compounds that were likely to reverse gene expression changes induced by MARV infection, the top 25 perturbagens with most negative connectivity scores were enalapril, GR-127935, KU-0063794, BRD-K14329163, westcort, KU-0063794, clinofibrate, AZ-628, carbenoxolone, trimetazidine, zolpidem, orlistat, K-858, PI-103, PP-2, letermovir, BRD-K95992530, ebselen, lucitanib, ranitidine, IOX2, selamectin, MEK-162, equilin, and efaproxiral.

## Discussion

The persistence of viruses in bat cells and populations is a complex and multifaceted phenomenon. The complexity of this issue is further compounded by the diversity of bat species and their ecological roles, which influence virus transmission dynamics and persistence. Ongoing field research focused on bat-borne emerging pathogens coupled with advancements in next-generation sequencing technologies is vital for uncovering broader implications of these viral reservoirs. The rapid onset and high transmission rates of MVD present unique challenges, especially given the sporadic and unpredictable nature of MARV outbreaks, posing challenges for conducting human efficacy trials of MARV vaccines and treatments^51^-^54^. Therefore, understanding factors driving dynamic patterns in the transmission of MARV between bats and humans is essential for preventing and controlling MVD. Compared to previous studies, our current findings indicated that ER stress signaling, DNA damage, apoptosis, and SCAP/SREBP signaling are significantly enriched pathways in the *in vitro* MARV-infected bat model. In contrast, viral infection signaling, pathways related to IFN-alpha/beta signaling and oxidative stress responses were notably upregulated in the *in vitro* MARV-infected human model. This clarifies key differences between humans, who suffer from severe flu-like symptoms in response to the viral infection, and *R. aegyptiacus*, which, as a well-adapted reservoir, shows no notable illness during MARV infections in either natural or experimental settings^5^. When comparing the two models, pathways related to complement signaling, IFN-alpha/beta signaling, WNT/Beta-catenin signaling, and HH signaling were identified as enriched pathways regulated by common leading edge genes under MARV infection conditions in both species. Targeting these pathways offers a promising opportunity to develop innovative treatments, supplementing current antiviral therapies and bringing new hope against the challenges of MARV.

Complement activation is a key component of the innate immune system, whose activation leads to the rapid clearance of viral pathogenesis in the early phase of infection^55^. In general, the complement system can be activated through three primary pathways: the antibody-induced classical pathway, a lectin pathway via mannose-binding lectin (MBL), and an antibody-independent alternative pathway^56^. Earlier studies established a connection between filoviral infection and the first two pathways, particularly the binding of MBL to envelope glycoproteins of EBOV and MARV, as reported by Ji *et al.* and Michelow *et al.*, which contributed to the development of antibody-based therapies^57^-^59^. To date, no research findings have yet linked the third pathway to MARV infection, but there is evidence of such a link with the influenza virus and Chandipura virus^60^, ^61^. Therefore, targeting alternative pathways, in addition to the lectin pathway, may offer new therapeutic strategies for combating viral infections. The relationship between IFN and viral replication is complex and involves a dynamic interplay between a host's immune defenses and the virus' strategies to evade them. Type I IFNs (IFN-α/β) are key components of the innate immune response. When a virus infects a host cell, the cell detects the presence of viral components (such as double-stranded RNA) and triggers the production of type I IFNs. IFN-α/β then bind to their receptors on the surface of both infected and neighboring cells, initiating a signaling cascade that leads to expressions of IFN-stimulated genes (ISGs). These genes produce proteins that inhibit various stages of viral replication, including viral entry, replication, and assembly. Many viruses, including filoviruses like EBOV and MARV, have evolved mechanisms to evade or suppress the IFN response. In 2003, a study of Bray *et al.* explored the role of the type I IFN (IFN-α/β) responses in protecting against filovirus infections, such as EBOV and MARV, and highlighted that mice lacking the IFN-α/β receptor or treated with antibodies against IFN-α/β could be lethally infected with EBOV or MARV^62^. More-recent studies, including those by Cao *et al.* and Basler *et al.*, demonstrated how filoviruses evade a host's IFN responses. Both studies focused on how viral proteins (VPs), particularly VP35 and VP24, interfere with IFN signaling pathways, allowing the virus to suppress immune responses, thereby facilitating robust viral replication and spread within the host^63^, ^64^. Thus, a strong and early IFN response can control viral replication, limiting the spread of the virus and reducing disease severity. In 2011, Guito *et al.* discovered differences in the potency of IFN antagonism among various filoviruses. They confirmed that VP35 is the primary antagonist of IFN production, while other VPs, such as VP24 and MARV VP40, are responsible for inhibiting downstream IFN signaling pathways across different filoviruses, including EBOV and MARV^65^. An *in silico* study by Alsaady *et al.* later identified six compounds—estradiol benzoate, paliperidone, isosilybin, protopanaxadiol, permethrin, and bufalin—as potential inhibitors of the MARV VP35 protein; however, further experimental validation is required to confirm their efficacy^66^.

The Wnt/β-catenin signaling pathway was reported to facilitate viral replication, notably in the case of severe acute respiratory syndrome (SARS)-coronavirus (CoV)-2^67^, ^68^. However, this pathway has shown opposing roles, either promoting or inhibiting viral replication, as observed with bovine parainfluenza virus type, herpes simplex virus, and pseudorabies virus^69^-^72^. In the context of filoviruses, RNA-Seq results of MARV-Angola variant-infected macaques that were pre-exposed to recombinant vesicular stomatitis virus (rVSV) vectors expressing the MARV-Angola-glycoprotein (GP) (Woolsey *et al.*) confirmed upregulation of the Wnt signaling pathway in survivors compared to those that died^73^. Recently, RNA-Seq results of human monocyte-derived macrophages inoculated with bat-isolated MARV repeatedly confirmed upregulation of the Wnt/β-catenin pathways in MARV-infected groups compared to both the lipopolysaccharide (LPS)-treated (positive control) and mock-infected (negative control) groups^74^. These consistent research findings once again highlight the significant role of Wnt signaling pathways, particularly in the case of MARV infection. These pathways could be targeted for drug development or used in treatment evaluation, in addition to the use of IFNs as discussed above. HH signaling refers to key pathways regulated by the *HH* gene family, which includes the secreted proteins Sonic HH (SHH), Indian HH (IHH), and Desert HH (DHH) in mammals. Dysregulated HH signaling was previously reported to result in developmental disorders and cancer. However, specific functions of this pathway in immunomodulation and viral infection have not been thoroughly explored^75^. Kim *et al.*, Yang *et al.*, and Arzumanyan *et al.* reported enrichment of the HH signaling pathway in heaptitis B virus (HBV) infection; Singh *et al.* highlighted the relationship between the disruption of HH signaling in the central nervous system (CNS) and human immunodeficiency virus (HIV)-associated neurocognitive disorder (HAND); Thawani *et al.* explored implications of HH signaling in Zika virus (ZIKV)-induced microcephaly, while other previous findings reported the relationships between this signaling pathway and oncoviruses^76^-^81^. Existing evidence suggests that impairing various cellular pathways related to the cell cycle, DNA damage repair mechanisms, and cellular metabolism can lead to uncontrolled cell growth, the accumulation of genetic mutations, and metabolic reprogramming, all of which are hallmarks of cancer. Notably, impaired HH, Notch, and WNT signaling pathways are significantly associated with oncoviral infection. To date, eight major human viruses have been linked to oncogenesis and are therefore referred to as oncoviruses. These include human T-cell lymphotropic virus type I (HTLV-1), hepatitis C virus (HCV), HBV, Merkel cell polyomavirus (MCPV), human cytomegalovirus (CMV), human papillomavirus (HPV), Epstein-Barr virus (EBV), and Kaposi's sarcoma-associated herpesvirus (KSHV)^82^, ^83^. So far, no research has established a link between the MARV and hallmarks of cancer, nor has there been any examination of the connection between HH signaling and MARV infection. These areas appear to be unexplored and could benefit from further investigation.

In the context of drug discovery, among the top five compounds with the most negative connectivity scores, enalapril ranked first. Enalapril is a prodrug belonging to the angiotensin-converting enzyme (ACE) inhibitor class that targets the renin-angiotensin-aldosterone system-a key regulator of blood pressure and fluid-electrolyte balance. Its active metabolite, enalaprilat, inhibits the ACE, reducing angiotensin II production, which decreases vasoconstriction and sodium reabsorption, ultimately lowering blood pressure and fluid volume^84^. Significant previous findings hightlighted enalapril as a potential compound related to drug repurposing against dengue virus and chikungunya virus^85^, ^86^. Notably, a significant number of previous findings also indicated that the ACE, being predominantly expressed by proximal tubules and glomerular podocytes, is a key receptor for SARS-CoV-2 entry into human cells via interaction with its spike (S) protein. This contributes to early kidney involvement during the course of infection, which is associated with worse outcomes in SARS-CoV-2 patients^87^-^89^. Although past systematic reviews and meta-analyses showed conflicting results regarding the use of ACE inhibitors and outcomes in SARS-CoV-2 patients, subsequent *in silico*, *in vitro,* and *in vivo* approaches demonstrated promising results^90^-^93^. Remarkably, a RNA-Seq analysis of livers from bats infected with EBOV and MARV (raw data deposited at GSE152728) revealed decreased expression of angiotensinogen (AGT) but increased expression of prostaglandin I2 synthase (PTGIS), resulting in elevated ACE levels, all of which are blood pressure-regulating genes^39^. Other field studies confirmed the improved outcomes of EBRV-infected inpatients treated with angiotensin receptor blockers (ARBs), namely Irbesartan, as reported from an observational study^94^. The WHO also lists ACE inhibitors in their clinical management standard operating procedures (SOPs) for EBOV disease, suggeting that enalapril can serve as a highly promising candidate for treating filovirus infections in general^95^. The compound GR127935, which ranked second, is a 5-hydroxytryptamine (5-HT) receptor antagonist that primarily binds to the 5-HT1A, 5-HT2A, and 5-HT7 receptors and has been widely studied in neurological disease research. Various studies, including those by Hu *et al.* and Riva *et al.*, underscored its potential against both the HCV and SARS-CoV-2^96^, ^97^. Additionally, Ahmad *et al.*'s *in silico* drug repurposing study identified GR127935 as one of the top scoring compounds targeting the ACE2 host-virus interface, highlighting its promise in blocking SARS-CoV-2 entry^98^. Another target, mammalian targe or rapamycin (mTOR), has become increasingly recognized for its potential as a target in antiviral therapies. KU0063794 is a selective inhibitor of the mTOR, a key pathway that plays a crucial role in regulating cell growth, proliferation, and survival. mTOR inhibitors can interfere with a virus's ability to manipulate host cell processes, thereby hindering viral replication, making mTOR a promising candidate in antiviral treatment strategies. In summary, although the aforementioned compounds and targets show potential, no studies to date have evaluated their antiviral efficacy against MARV infection. Further experimental studies are necessary to confirm these findings.

Additionally, our screening results identified several potential targets, although the supporting evidence is currently limited and requires further validation to confirm their effectiveness in antiviral therapies. The identified targets included acetylcholine receptor antagonists (biperiden and naphazoline), adrenergic receptor agonists (reboxetine and urapidil), cyclooxygenase inhibitors (nefopam, valdecoxib, and ebselen), epidermal growth factor receptor (EGFR) inhibitors (WZ-4002 and lapatinib), fibroblast growth factor receptor (FGFR) inhibitors (AZD-4547 and lucitanib), histamine receptor agonists (ranitidine, doxylamine, and SNX-2112), heat shock protein (HSP) inhibitors (SNX-2112 and BIIB-021), insulin-like growth factor (IGF)-1 inhibitors (BMS-536924 and linsitinib), lipase inhibitors (clinofibrate and orlistat), MAPK kinase (MEK) inhibitors (MEK-162, U-0126, TAK-733, selumetinib, AS-703026, PD-98059, and PD-0325901), phosphatidylinositol 3 kinase (PI3K) inhibitors (AS-605240, GDC-0941, and PI-103), RAF inhibitors (AZ-628, vemurafenib, and AZ-628), Rho-associated kinase inhibitors (BRD-K23875128, fasudil, and GR-127935), an interleukin receptor agonist (pidotimod), a DNA inhibitor (pibenzimol), an estrogen receptor agonist (equilin), and a glucocorticoid receptor agonist (westcort). Further research is needed to fully explore and validate the potential of these compounds in antiviral therapies. The original study by Arnold *et al.* (GSE117367) found that the Egyptian rousette bat serves as a natural reservoir for MARV and effectively controls viral infections with low viremia and no disease symptoms, unlike humans who suffer severe disease due to MARV-induced IFN antagonism. Analysis of the ERB genome and transcriptome revealed that while MARV inhibited antiviral responses in ERB cells, it did not induce specific IFN genes. However, there were increased expressions of unannotated antiviral genes and higher basal expressions of key antiviral genes in uninfected ERB cells. In contrast, findings of Koch *et al.* (GSE226148) highlighted the impact of MARV infection on human primary proximal tubular cells (PTCs). They observed a significant inflammatory response characterized by the activation of signaling pathways associated with IFNs (IFN-α and IFN-y) and tumor necrosis factor (TNF)-α, along with downregulation of gene sets related to energy metabolism and mitochondrial function, which may contribute to the acute kidney injury observed in MVD. In our current study, by analyzing common DEGs between the two studies, we discovered that these genes are primarily associated with pathways related to the complement system, the innate immune response involving IFNs, Wnt/β-catenin signaling, and HH signaling, all of which appeared to play significant roles in the context of MARV infection in both models. Additionally, our analysis identified several potential compounds, notably the ACE inhibitor, enalapril, that could be used to combat MARV infection.

As the first-ever report of its kind, this study provides valuable insights into the underlying mechanisms of MARV's pathophysiology and offers suggestions regarding modes of transmission, post-infection consequences, and potential avenues for future vaccine development. The findings represent a significant step in bridging the gap between laboratory research and clinical applications, a crucial aspect in the rapid development of effective treatment strategies for MARV outbreaks. Additionally, the outcomes of our study offer critical guidance for experimental efforts aimed at developing vaccines against MARV, contributing to the ongoing global efforts to combat the potential threat of MARV outbreaks. The primary limitation of this study was its reliance on *in vitro* models, specifically MARV-infected bat and human cell lines. While these models offer valuable insights into cellular responses to viral infection, they do not fully replicate the complex immune and systemic dynamics of a whole organism. *In vitro* systems are inherently limited in mimicking intricate interactions among different cell types, tissues, and the immune system. Consequently, this can lead to an overrepresentation of systemic pathways, which might not be as prominently activated during localized or tissue-specific responses observed in actual viral infections *in vivo*. As a result, the study's findings, particularly regarding activation of certain immune or signaling pathways, might not fully reflect localized responses seen in living organisms. Furthermore, because the study did not account for the immune system's role in viral clearance or drug efficacy, the identified pathways and compounds should be validated in more-complex models that better mimic human physiology, such as animal models or *ex vivo* tissue systems, before progressing to clinical trials.

## Supplementary Material

Supplementary figures and tables.
