# Supplementary material for: A comparative analysis of Marburg virus-infected bat and human models from public high-throughput sequencing data
Source: Int J Med Sci. 2025 Jan 1;22(1):1–16. doi: 10.7150/ijms.100696 (PMC11659840; doi:10.7150/ijms.100696)
Supplement: Supplementary file 1 — Supplementary figures and tables. [file ijmsv22p0001s1.pdf]

Supplementary Materials

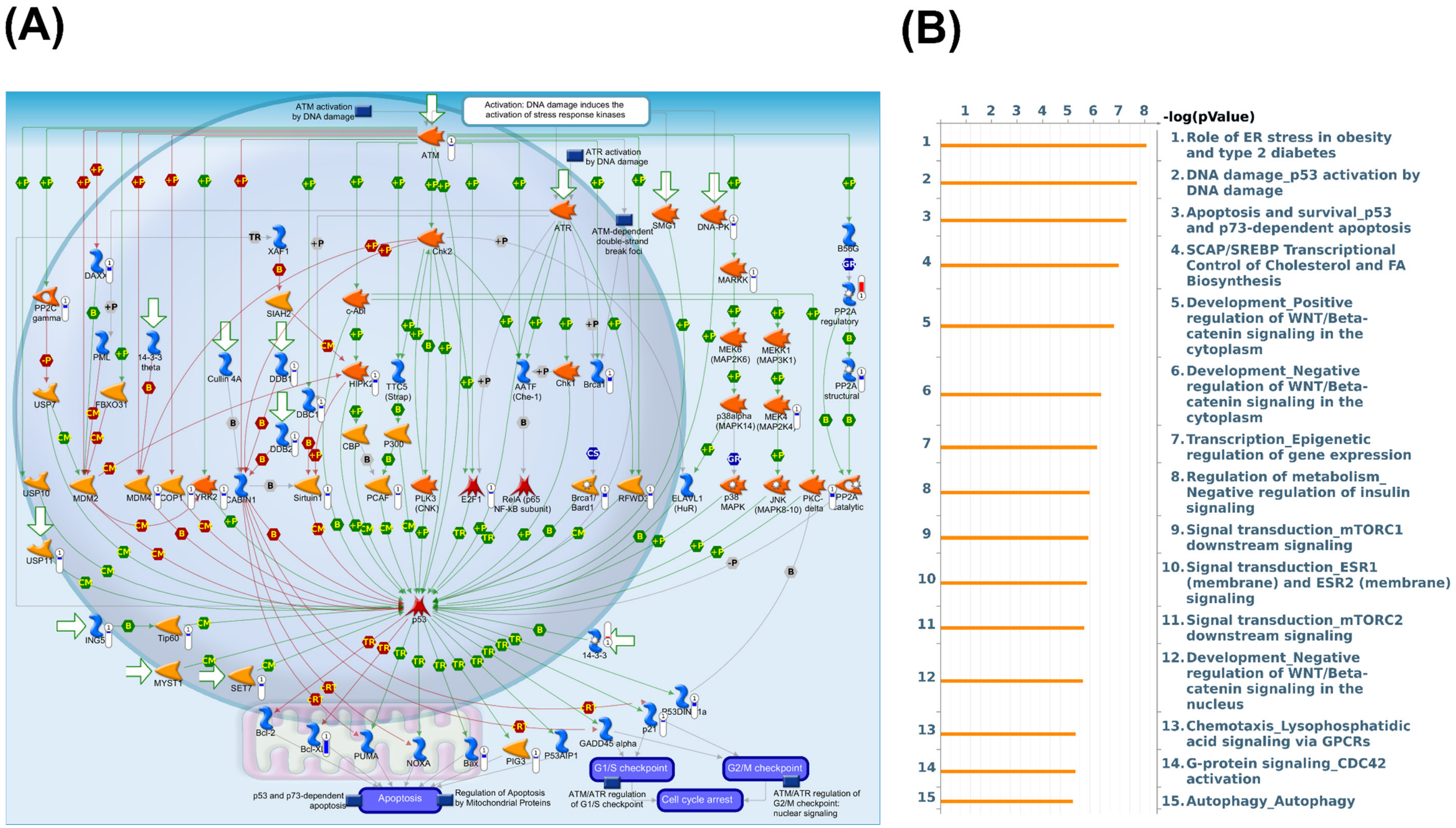

Supplementary Figure 1. Differentially expressed gene (DEG)-enriched signaling pathways regulated by the top upregulated genes of a Marburg virus (MARV)-infected *Rousettus aegyptiacus* model compared to mock-infected controls. (A) List of the top 20 DEG-enriched signaling pathways associated to the top upregulated genes within the GSE117367 dataset, generated by MetaCore. (B) Visualization of the global signal transduction pathway network generated by MetaCore confirmed the key role of the "DNA damage_p53 activation by DNA damage" in the MARV-infected *Rousettus aegyptiacus* model.

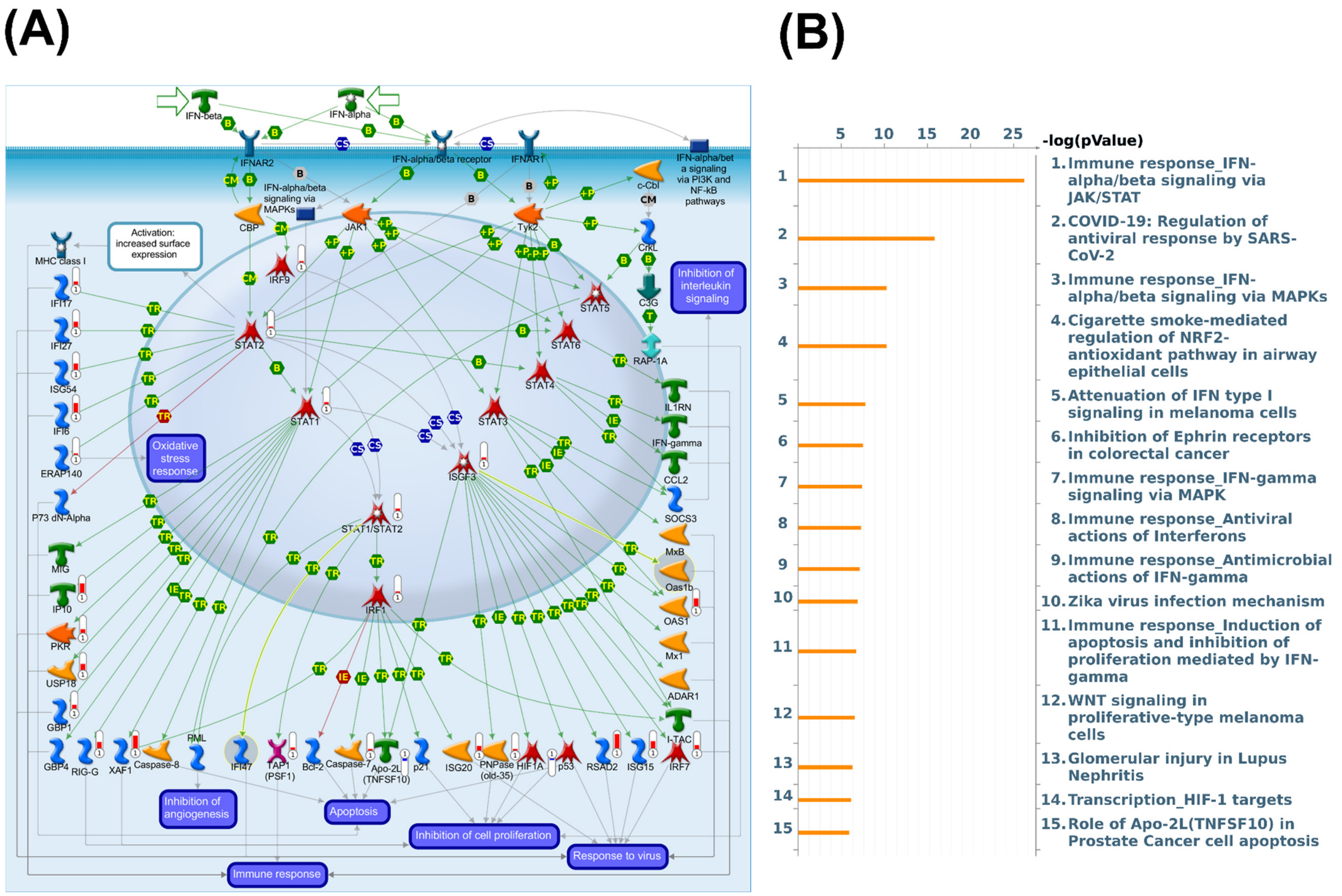

Supplementary Figure 2. Differentially expressed gene (DEG)-enriched signaling pathways regulated by the top upregulated genes in a Marburg virus (MARV)-infected human model compared to mock-infected controls. (A) List of the top 20 DEG-enriched signaling pathways associated with the top upregulated genes within the GSE226148 dataset, generated by MetaCore. (B) Visualization of the global signal transduction pathways network generated by MetaCore confirmed the key role of the “Immune response_IFN-alpha/beta signaling via JAK/STAT” in the MARV-infected human model.

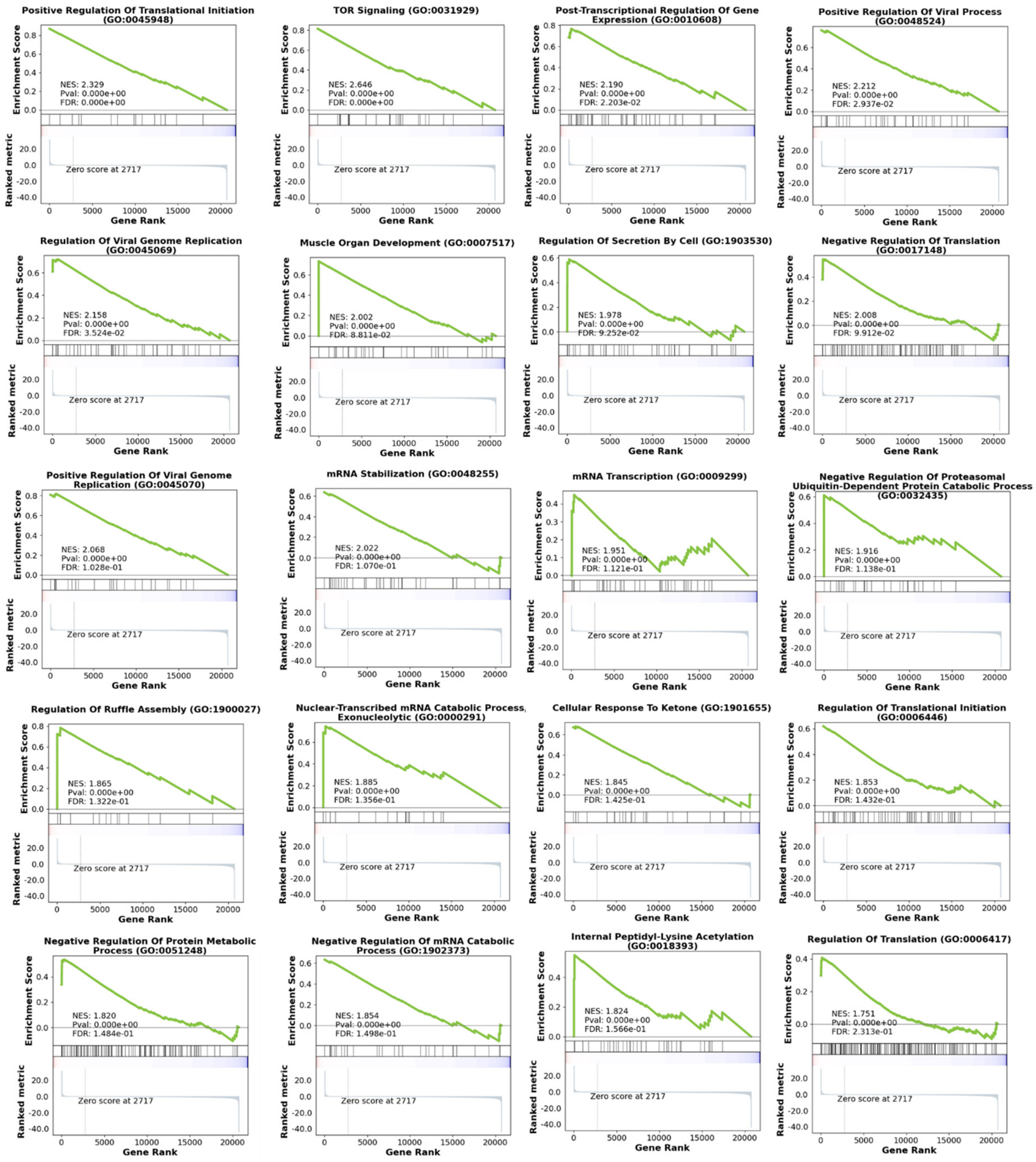

Supplementary Figure 3. Enriched pathways in Marburg virus (MARV)-infected *Rousettus aegyptiacus in vitro* model. The listed differentially expressed gene (DEG)-enriched signaling pathways associated with 3212 differentially regulated genes in the GSE117367 dataset included Positive Regulation of Translational Initiation, TOR Signaling, Post-Transcriptional Regulation of Gene Expression, Positive Regulation of Viral Processes, Regulation of Viral Genome Replication, Muscle Organ Development, Regulation of Secretion By Cells, Negative Regulation of Translation, Positive Regulation of Viral Genomse, mRNA Stabilization, mRNA Transcription, Negative Regulation of Proteasomal Ubiquitin-Dependent Protein Catabolic Processes, Regulation of Ruffle Assembly, Nuclear-Transcribed mRNA Catabolic Processes, Cellular Responses to Ketone, Regulation of Translational Initiation, Negative Regulation of Protein Metabolic Processes, Negative Regulation of mRNA Catabolic Processes, Internal Peptidyl-Lysine Acetylation, and Regulation of Translation.

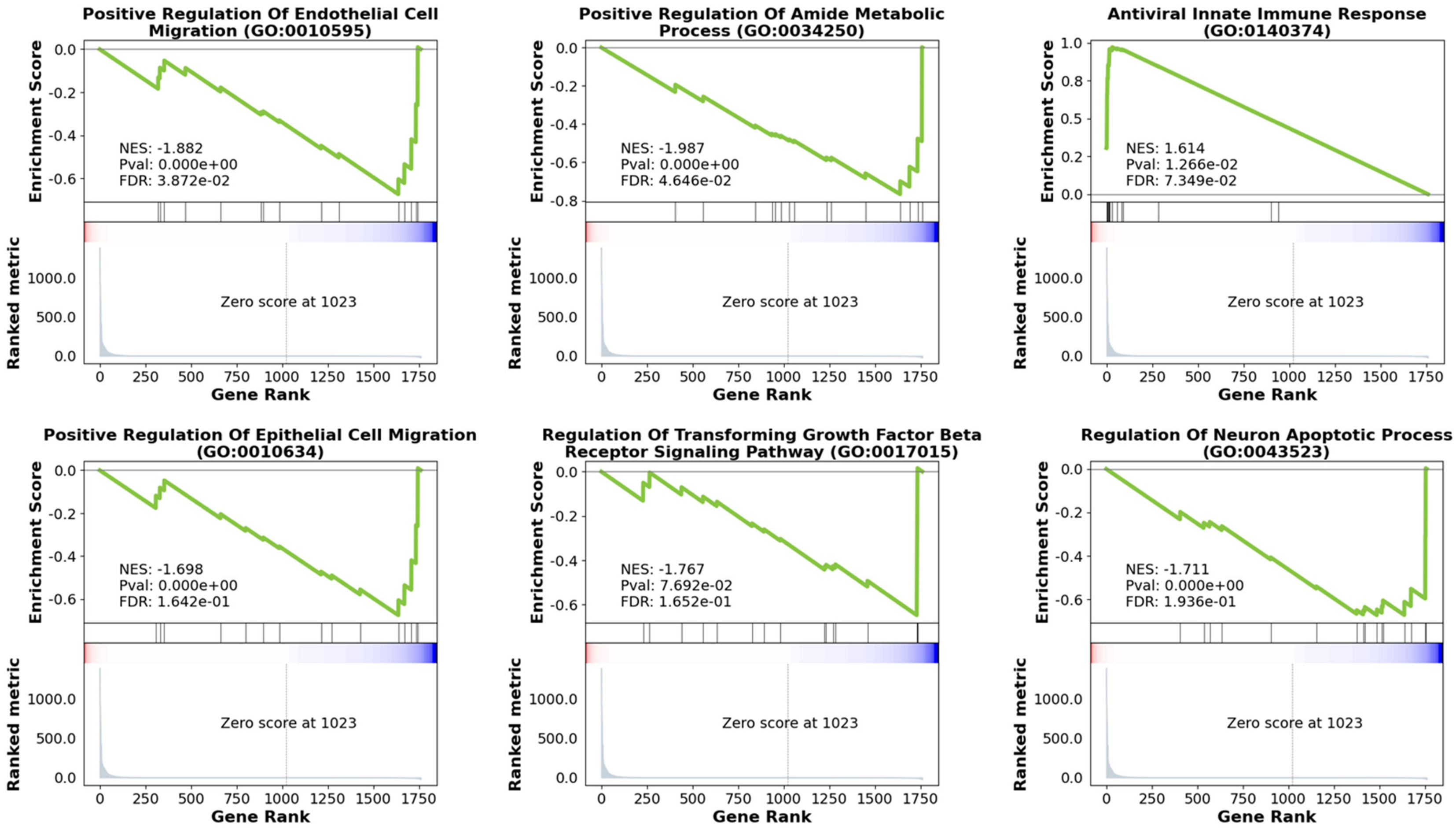

Supplementary Figure 4. Enriched pathways in a Marburg virus (MARV)-infected human *in vitro* model. The listed differentially expressed gene (DEG)-enriched signaling pathways associated with 682 differentially regulated genes in the GSE226148 dataset included the Positive Regulation of Endothelial Cell Migration, Positive Regulation of Amide Metabolism, Antiviral Innate Immune Responses, Positive Regulation of Epithelial Cell Migration, Regulation of the Transforming Growth Factor-Beta Receptor Signaling Pathway, and Regulation of the Neuron Apoptotic Processes.

Supplementary Table A1. List of common differentially expressed genes (DEGs) shared by the GSE117367 dataset (MARV-infected *Rousettus aegyptiacus in vitro* model) and the GSE226148 dataset (MARV-infected human *in vitro* model), with a log fold change cutoff of > 1.5 and a *p* value cutoff of < 0.5.

No	Gene Symbol	GSE117367		GSE226148	
		log2FoldChange	p-value	log2FoldChange	p-value
1	ADAMTS12	-0.6521	0.0019	0.9565	0.0256
2	ADM	1.0196	0.0152	1.4885	0.0000
3	AHR	-1.5042	0.0001	0.9485	0.0000
4	ARHGAP39	-0.8665	0.0451	-0.6229	0.0347
5	ATXN1	-0.9517	0.0310	0.7168	0.0000
6	B3GNT5	-0.7011	0.0117	0.6388	0.0009
7	CA12	-1.1170	0.0211	1.6958	0.0000
8	CASP7	-0.8690	0.0082	0.7900	0.0000
9	CD274	-0.6575	0.0145	1.9646	0.0000
10	CDKL1	-0.7054	0.0263	0.6040	0.0031
11	CDKN1C	-1.1838	0.0074	-1.4443	0.0000
12	CFB	-1.1517	0.0012	0.6741	0.0013
13	CPEB3	-1.0648	0.0017	0.6999	0.0224
14	DAB2	-0.7564	0.0222	-0.6792	0.0000
15	DNAJB4	-0.7029	0.0097	0.8916	0.0000
16	DNMBP	-0.7328	0.0430	0.5959	0.0003
17	DTX3L	-0.7514	0.0063	1.2451	0.0000
18	DUSP10	1.1813	0.0470	0.8093	0.0002
19	DUSP4	0.8822	0.0005	1.3992	0.0000
20	DUSP5	0.6015	0.0298	1.3461	0.0000
21	DYNC2LI1	-0.8916	0.0027	-0.6446	0.0000
22	E2F7	-0.7060	0.0103	0.5905	0.0083
23	EDN2	0.7513	0.0071	-0.8805	0.0000
24	FAM161B	-0.6961	0.0021	-0.6684	0.0228
25	FGFR2	-0.7783	0.0239	-0.8049	0.0001
26	FHL1	-0.8791	0.0309	-0.7322	0.0000
27	FILIP1L	-0.9883	0.0008	-1.0501	0.0000
28	FOSB	-0.6012	0.0366	1.1351	0.0324
29	FRAS1	-1.1462	0.0003	0.6154	0.0051
30	FREM2	-0.7241	0.0261	0.6184	0.0072
31	FZD5	-0.7513	0.0020	0.6351	0.0000
32	GCH1	-0.8058	0.0158	0.7111	0.0018
33	GJB3	0.7074	0.0105	1.5239	0.0041
34	GLI4	-0.6156	0.0347	-0.7030	0.0380
35	GPAT3	-0.7130	0.0173	1.5920	0.0000
36	GPR180	-0.6086	0.0221	0.7429	0.0000
37	GPSM1	-0.7845	0.0144	-0.7101	0.0038
38	HDAC9	-1.1014	0.0240	0.9939	0.0000
39	HEG1	-0.5731	0.0086	0.8907	0.0000
40	HERC5	-1.2492	0.0125	4.5211	0.0000
41	IFIH1	-0.7306	0.0130	3.3761	0.0000

**42**	INPP1	-0.9893	0.0179	0.6896	0.0085
**43**	IRF9	-0.7868	0.0044	0.7081	0.0000
**44**	LAMB3	-0.3515	0.0288	1.4220	0.0115
**45**	LDLR	-0.5955	0.0045	1.3074	0.0045
**46**	LIN52	-0.8354	0.0271	0.6869	0.0238
**47**	LYST	-0.6251	0.0032	0.6376	0.0003
**48**	MDM4	-0.8122	0.0258	0.7470	0.0000
**49**	MYCL	-1.2069	0.0005	-1.1537	0.0275
**50**	NCOA7	-1.2155	0.0210	0.5967	0.0002
**51**	NEIL3	-0.6147	0.0164	0.9060	0.0001
**52**	NOCT	-0.6097	0.0140	1.2243	0.0000
**53**	NRP1	-0.7180	0.0121	-0.6692	0.0000
**54**	NUB1	-0.6329	0.0015	0.6564	0.0000
**55**	PAQR5	-0.9615	0.0022	0.6025	0.0070
**56**	PDZD2	-0.7553	0.0216	0.8222	0.0469
**57**	PHACTR2	-0.5865	0.0161	0.6274	0.0000
**58**	PIK3IP1	-0.6515	0.0053	-0.9942	0.0001
**59**	PLCH1	-1.1966	0.0007	0.8444	0.0116
**60**	PLEKHA4	-0.7607	0.0148	0.6457	0.0005
**61**	PPM1D	-0.6403	0.0020	-0.7846	0.0000
**62**	PXK	-1.5949	0.0080	0.7592	0.0024
**63**	RAB9B	-0.9936	0.0020	-0.6003	0.0260
**64**	RASL11B	-0.6280	0.0098	-1.7309	0.0060
**65**	RNF213	-13.0904	0.0004	1.5961	0.0000
**66**	RPS6KA6	-0.8106	0.0161	0.8809	0.0018
**67**	SBK1	-0.7662	0.0115	-0.8741	0.0078
**68**	SHE	-0.7047	0.0100	0.9908	0.0007
**69**	SLC25A28	-0.6842	0.0093	0.5852	0.0030
**70**	SLC26A1	-0.5989	0.0298	0.7088	0.0112
**71**	SLC43A2	-1.1994	0.0037	-1.0072	0.0002
**72**	SLC6A6	-0.8602	0.0008	-0.8122	0.0000
**73**	SMO	-0.5960	0.0160	-0.6331	0.0062
**74**	SOX4	-0.6891	0.0031	-0.9884	0.0000
**75**	SP100	-0.8646	0.0192	1.1186	0.0000
**76**	SPP1	-0.7556	0.0051	0.6272	0.0000
**77**	SSBP2	-0.6610	0.0236	-0.7057	0.0468
**78**	STARD4	-0.6835	0.0411	1.9804	0.0000
**79**	STAT2	-0.7840	0.0018	0.8757	0.0000
**80**	SUFU	-0.6894	0.0083	-0.6570	0.0000
**81**	TMEM170A	-0.6119	0.0133	0.6001	0.0003
**82**	TMEM86A	-0.7379	0.0217	-1.0007	0.0473
**83**	TMEM87B	-1.2824	0.0210	0.6277	0.0000
**84**	TRIM34	-0.9638	0.0073	0.9677	0.0062
**85**	TRIM45	-0.4633	0.0368	-0.9603	0.0004
**86**	TRIM6	-0.6066	0.0029	0.6783	0.0000
**87**	TSPAN33	-0.8956	0.0464	-0.8343	0.0059
**88**	TTLL7	-0.6117	0.0185	0.6889	0.0000
**89**	ZBTB38	-1.1248	0.0226	-0.5983	0.0000
**90**	ZNF557	-0.6840	0.0046	0.8442	0.0001

Supplementary Table A2. Lists of gene ontology (GO) terms of biological processes of the GSE117367 dataset (Marburg virus (MARV)-infected *Rousettus aegyptiacus in vitro* model) and GSE226148 dataset (MARV-infected human *in vitro* model), with a log fold change cutoff of > 1.5 and a *p* value cutoff of < 0.5.

GSE117367

ID	Description	GeneRatio	BgRatio	-log(pvalue)
**GO:0016570**	histone modification	35/612	468/18866	5.3632
**GO:0016569**	covalent chromatin modification	35/612	481/18866	5.1029
**GO:0032933**	SREBP signaling pathway	5/612	14/18866	4.2560
**GO:0071501**	cellular response to sterol depletion	5/612	15/18866	4.0916
**GO:0006991**	response to sterol depletion	5/612	17/18866	3.8011
**GO:0070507**	regulation of microtubule cytoskeleton organization	17/612	190/18866	3.7996
**GO:0010506**	regulation of autophagy	25/612	347/18866	3.7457
**GO:0098732**	macromolecule deacylation	12/612	111/18866	3.5888
**GO:0051494**	negative regulation of cytoskeleton organization	15/612	163/18866	3.5524

GSE226148

ID	Description	GeneRatio	BgRatio	-log(pvalue)
**GO:0051607**	defense response to virus	34/208	258/18866	26.1305
**GO:0034340**	response to type I interferon	24/208	99/18866	25.1003
**GO:0060337**	type I interferon signaling pathway	23/208	95/18866	24.0491
**GO:0071357**	cellular response to type I interferon	23/208	95/18866	24.0491
**GO:0009615**	response to virus	36/208	349/18866	23.9200
**GO:0045071**	negative regulation of viral genome replication	16/208	61/18866	17.4574
**GO:0048525**	negative regulation of viral process	18/208	104/18866	16.0990
**GO:0045069**	regulation of viral genome replication	17/208	100/18866	15.0922
**GO:1903901**	negative regulation of viral life cycle	16/208	86/18866	14.8838

Supplementary Table A3. Lists of 50 differentially expressed gene (DEG)-enriched signaling pathways shared between two Marburg virus (MARV)-infected models. The listed DEG-enriched signaling pathways were associated with 90 DEGs shared by the GSE117367 dataset (MARV-infected *Rousettus aegyptiacus in vitro* model) and GSE226148 dataset (MARV-infected human *in vitro* model), generated by MetaCore, and sorted in descending order of log(*p* values).

#	Maps	p-value	Network Objects from Active Data
1	Alternative complement cascade disruption in age-related macular degeneration (AMD)	7.814E-06	Factor H, Factor Ba, Factor Bb, Factor B
2	Immune response_Alternative complement pathway	6.794E-05	Factor H, Factor Ba, Factor Bb, Factor B
3	Immune response_IFN-alpha/beta signaling via JAK/STAT	1.259E-04	IRF9, Caspase-7, ERAP140, STAT2
4	SHH signaling in colorectal cancer	2.835E-04	Frizzled, Smoothened, SUFU
5	Development_Embryonal epaxial myogenesis	2.835E-04	Frizzled, Smoothened, SUFU
6	SHH signaling in melanoma	3.420E-04	Smoothened, SUFU, Osteopontin
7	Development_Early embryonal hypaxial myogenesis	4.814E-04	Frizzled, Smoothened, SUFU
8	SHH signaling in oligodendrocyte precursor cells differentiation in multiple sclerosis	1.292E-03	Smoothened, SUFU
9	Stem cells_Aberrant Hedgehog signaling in medulloblastoma stem cells	1.729E-03	Smoothened, SUFU
10	Development_Positive regulation of WNT/Beta-catenin signaling at the receptor level	2.384E-03	Frizzled, FGFR2, FZD5
11	Development_Positive regulation of WNT/Beta-catenin signaling in the nucleus	2.955E-03	Frizzled, SOX4, WIP1
12	Stellate cells activation and liver fibrosis	3.078E-03	DAB2, Frizzled, Smoothened
13	Immune response_IFN-alpha/beta signaling via MAPKs	3.467E-03	TRIM6, IRF9, GCH1
14	Stem cells_Aberrant Wnt signaling in medulloblastoma stem cells	4.079E-03	DAB2, Frizzled
15	Hedgehog signaling in breast cancer	4.438E-03	Smoothened, Osteopontin
16	Development_Negative regulation of WNT/Beta-catenin signaling in the cytoplasm	4.645E-03	Porf-2, DAB2, Frizzled
17	Putative pathways of hormone action in neurofibromatosis type 1	4.811E-03	FosB, Adrenomedullin
18	Transport_Intracellular cholesterol transport	4.973E-03	DAB2, StARD4, LDLR
19	Hedgehog signaling in prostate cancer	5.198E-03	Smoothened, SUFU
20	Tumor-stroma interactions in pancreatic cancer	6.014E-03	Smoothened, SUFU
21	Hedgehog signaling in gastric cancer	6.442E-03	Smoothened, SUFU
22	Transport_Cholesterol and Sphingolipid influx to the early endosome in lung	6.442E-03	DAB2, LDLR
23	Bone metastases in Prostate Cancer	6.442E-03	Frizzled, Osteopontin

**24**	Development_WNT/Beta-catenin signaling in organogenesis	7.339E-03	Frizzled, Neuropilin-1
**25**	Hedgehog signaling in pancreatic cancer	7.808E-03	Smoothened, SUFU
**26**	SLE genetic marker-specific pathways in B cells	8.102E-03	IRF9, PXK, STAT2
**27**	Role of SHH and Notch in small cell lung cancer (SCLC)	8.289E-03	Smoothened, SUFU
**28**	Development_EGF-induced proliferation of Type C cells in SVZ of adult brain	8.289E-03	Smoothened, SUFU
**29**	Endothelial cell apoptosis in COPD	9.812E-03	Neuropilin-1, Caspase-7
**30**	E-cadherin signaling and its regulation in gastric cancer	9.812E-03	Frizzled, FGFR2
**31**	Attenuation of IFN type I signaling in melanoma cells	1.035E-02	IRF9, STAT2
**32**	Transport_Cholesterol and Sphingolipid influx to the early endosome in liver	1.089E-02	DAB2, LDLR
**33**	Congenital Zika Syndrome	1.202E-02	Frem2, Caspase-7
**34**	Stem cells_Inhibition of Hedgehog signaling in medulloblastoma stem cells	1.260E-02	FGFR2, Smoothened
**35**	Epigenetic alterations in survival and migration of small cell lung cancer (SCLC) cells	1.381E-02	DAB2, LAMB3
**36**	Development_Regulation of lung epithelial progenitor cell differentiation	1.381E-02	Frizzled, FGFR2
**37**	Zika virus infection mechanism	1.506E-02	MDA-5, STAT2
**38**	Development_Negative regulation of WNT/Beta-catenin signaling at the receptor level	1.506E-02	DAB2, Frizzled
**39**	Dual function of Treg cells in cancer development	1.570E-02	Neuropilin-1, STAT2
**40**	Immune response_Induction of apoptosis and inhibition of proliferation mediated by IFN-gamma	1.635E-02	Caspase-7, STAT2
**41**	Immune response_IFN-gamma signaling via MAPK	1.908E-02	SP100, IRF9
**42**	Development_Induction of chondrogenesis and ossification by NOTCH signaling	1.980E-02	p57, Osteopontin
**43**	Immune response_Antiviral actions of Interferons	1.980E-02	IRF9, STAT2
**44**	Immune response_IL-5 signaling via JAK/STAT	2.275E-02	DUSP5, SOX4
**45**	Vascular endothelial cell damage in SLE	2.832E-02	PD-L1, Caspase-7
**46**	Neuroprotective action of lithium	2.832E-02	Frizzled, FZD5
**47**	Ligand-independent activation of Androgen receptor in Prostate Cancer	3.173E-02	Frizzled, FGFR2
**48**	Reproduction_Gonadotropin-releasing hormone (GnRH) signaling	3.712E-02	FosB, MKP-2
**49**	Signal transduction_mTORC1 upstream signaling	3.805E-02	MAG1, Frizzled
**50**	WNT signaling in the progression of lung cancer	4.090E-02	Frizzled, FZD5

Supplementary Table A4. Lists of 50 differentially expressed gene (DEG)-enriched signaling pathways within the Marburg virus (MARV)-infected MARV-infected *Rousettus aegyptiacus in vitro* model. The listed DEG-enriched signaling pathways were associated with 3212 differentially regulated genes within the GSE117367 dataset at MARV post-infection, generated by MetaCore, and sorted in descending order of log(*p* values).

#	Maps	p-value	Network Objects from Active Data
1	Development_Positive regulation of WNT/Beta-catenin signaling in the nucleus	7.525E-16	Casein kinase II, alpha chains, SOX11, TBL1X, DP1, Tcf(Lef), PIAS4, Alpha-1 catenin, TCF7L2 (TCF4), FOXP1, FOXM1, USP5, Jade-1, Beta-catenin, BCL9/B9L, VCP, Pin1, NCOA2 (GRIP1/TIF2), TDG, UBR5, NLK, Sirtuin1, GSK3 beta, CARF, HDAC2, FHL2, WIP1, PCAF, RUNX, WNT, FOXO3A, HMGB2, TBLR1, SOX4, HDAC1, APPL, ERK2 (MAPK1), LRRFIP2, SOX9, TCF7 (TCF1), Frizzled
2	Development_Negative regulation of WNT/Beta-catenin signaling in the nucleus	6.757E-14	KDM2, TBL1X, TRRAP, Calcineurin A (catalytic), WWOX, c-Cbl, BACH1, HBP1, PKC-delta, Tcf(Lef), E2F1, APC protein, Alpha-1 catenin, TCF7L2 (TCF4), 14-3-3, Jade-1, Beta-catenin, BCL9/B9L, PC1-CTT, CHD8, Menin, NF-AT5, NLK, GSK3 beta, Nephrocystin-4, HDAC2, WNT, FOXO3A, GPX4, P15RS, CHIBBY, RANBP3, TBLR1, HDAC1, TAB2, Plakoglobin, PJA2, HIC5, PPAR-gamma, Axin, TAK1(MAP3K7), SOX9, TCF7 (TCF1), Frizzled
3	Cell cycle_DNA replication initiation	7.550E-14	MCM3, RIF1, Cyclin B, CDC7, MCM complex, ORC1L, Histone H3, GINS2, MCM5, MCM4, E2F1, MCM10, Jade-1, Importin (karyopherin)-alpha, ORC6L, POLA1, TOPBP1, Geminin, CDK2, WDHD1, CDC18L (CDC6), HBOA, MCM7, Treslin, MCM6, POLA2, HMGI/Y, SMARCA5, RPA1, ORC5L, MCM4/6/7 complex, MCM2, HP1 alpha, SUPT16H, RPA2, RFWD3, CDC45L
4	Protein folding and maturation_Amyloid precursor protein processing (schema)	1.049E-13	APP-P3, alphaAPPs, MMP-24, APP-C99, etaAPP alpha, Caspase-9, APP, APP-C31, APP-CTF delta-short, APP-CTF delta-long, betaAPPs, ADAM9, deltaAPPs-80kD, etaAPP beta, APP-CTF theta, Caspase-3, BACE2, APP-C83 (CTF), APP-NCas, Amyloid beta 40, Legumain, APP-CTF eta, thetaAPPs, Amyloid beta, ADAM10, etaAPPs, ADAM17, APP-C59 (AICD), deltaAPPs-130kD, APP-Jcasp, Amyloid beta 42
5	DNA damage_Intra S-phase checkpoint	3.835E-13	MCM3, RAD18, RIF1, Brca1, CDC7, Histone H3, MCM5, SMC1, DTL (hCdt2), MCM4, HUWE1, MCM10, DNA-PK, CDH1, Rad50, p21, TOPBP1, p53BP1, CDK2, PCNA, CDC18L (CDC6), HBOA, MLL1 (HRX), Mitotic cohesin complex, Sirtuin1, DDB2, MCM7, BRIP1, SMC3, MRN complex, PCAF, ATM, ASCIZ, MCM2, Claspin, Nibrin, FBXW11, RPA2, CDC45L

**6**	Cell cycle_DNA replication: elongation and termination	3.423E-12	RFC4, POLE1, MCM3, RAD18, TOP1, MCM5, MCM4, DERPC, VCP, FEN1, DNA ligase I, RFC5, RNaseh2b, TIPIN, TRIP, CDK2, Ubiquitin, WDHD1, PCNA, UBE1, MCM7, ATAD5, TOP2 alpha, MCM6, RFC1, UBC3B, RFC2, TIM, MCM2, RNASEH2A, POLE3 (YBL1), Claspin, DCC1, CDC45L
**7**	Regulation of metabolism_Negative regulation of insulin signaling	5.059E-11	RhoA, PI3K cat class IA, CLK2, PARD3, PI3K reg class IA (p85-alpha), GRB2, c-Cbl, IRS-2, ENPP1, PKC-delta, IRS-1, ERK1/2, PTPN2, PP2A regulatory, c-Raf-1, IKK (cat), FBX29, p70 S6 kinases, PI3K reg class IA (p85), GSK3 beta, p70 S6 kinase1, PR61beta, PTEN, PTPR-alpha, PKC-alpha, PP2A structural, Insulin receptor, IKK-beta, QIK, MEK1/2, OGT (GlcNAc transferase), Cullin7, SHIP, ERK2 (MAPK1), SHIP2, GRB10, mTOR
**8**	Development_Positive regulation of WNT/Beta-catenin signaling in the cytoplasm	6.137E-11	Casein kinase II, alpha chains, GSKIP, TBL1X, HECTD1, TGF-beta 1, BIG1, GRB2, IRS-2, HIPK2, IRS-1, Tcf(Lef), RIPK4, APC protein, Alpha-1 catenin, 14-3-3, Beta-catenin, PKA-reg type II (cAMP-dependent), TGIF, RNF220, USP47, ILK, Rac1, Tankyrases, SIAH1, USP9X, WNT, Insulin receptor, Jouberin, GSK3 alpha/beta, TBLR1, ERK2 (MAPK1), RNF146, Axin, PKA-cat (cAMP-dependent), Frizzled, SET7
**9**	DNA damage_Double-strand break repair via homologous recombination	1.010E-10	RAD18, RIF1, DNA polymerase zeta, Brca1, Brca2, STARING, PALB2, MCM8, RNF168, FBH1, RMI2, TOPBP1, NUCKS, Rad51, p53BP1, CDK2, WDHD1, Ku80, DSS1, SAMHD1, SWI5, MRE11, BRIP1, MRN complex, RAD54B, RAD54L, ATM, Casein kinase II, RNF8, PPP4C, SMARCA5, Brca1/Bard1, RPA1, RBBP8 (CtIP), Nibrin, RNF20, RAP80, USP4
**10**	Apoptosis and survival_p53 and p73-dependent apoptosis	1.010E-10	MDM4, NIX, OKL38, NOR1, Aif, Sin3A, iASPP, HIPK2, Glyoxalase II, PKC-delta, Apaf-1, ASPP1, E2F1, HUWE1, ASPP2, DAXX, Pin1, IL4RA, Bak, RanBPM, MM-1, Bax, Sirtuin1, Caspase-10, Caspase-7, PTEN, DIP, PCAF, ASAP, Caspase-3, FasR(CD95), Bcl-XL, HtrA2, PIG3, Mitofusin 2, Bim, DBC1, Bid
**11**	Development_Negative regulation of WNT/Beta-catenin signaling in the cytoplasm	1.256E-10	E-cadherin, HECTD1, c-Cbl, HIPK2, DP1, Tcf(Lef), E2F1, HUWE1, RIPK4, APC protein, Alpha-1 catenin, Laforin, FAF1, Beta-catenin, PI3K cat class III (Vps34), CXXC5, DAB2, Nucleoredoxin, RNF185, YAP1/TAZ, Rac1, WWP1, LATS1, Malin, SIAH1, PKC-alpha, Porf-2, WNT, Beclin 1, NEDD4L, GSK3 alpha/beta, Ankyrin-G, WDR26, Skp2/TrCP/FBXW, TAZ, Axin, Frizzled
**12**	DNA damage_ATM/ATR regulation of G2/M checkpoint: nuclear signaling	7.184E-10	Brca1, Cyclin B, Cyclin B2, hnRNP K, CDC25C, PALB2, GTSE1, DNA-PK, CDH1, HDAC6, BTG2, p21, CDC14b, p53BP1, CDK2, WDHD1, CDC18L (CDC6), 14-3-3 sigma, ATM, Ku70, DNMT1, RBBP8 (CtIP), Cyclin B1, Claspin, CEP164

**13**	DNA damage_p53 activation by DNA damage	8.731E-10	MDM4, Brca1, HIPK2, PKC-delta, E2F1, DNA-PK, DAXX, 14-3-3, PP2A regulatory, DDB1, MARKK, p21, P53DINP1a, ING5, DYRK2, MEK4(MAP2K4), Tip60, USP11, Bax, Sirtuin1, DDB2, PCAF, PP2A structural, ATM, Bcl-XL, COP1, PIG3, Brca1/Bard1, DBC1, PP2C gamma, SET7, RFWD3
**14**	Protein folding and maturation_Regulation of amyloid precursor protein processing	8.999E-10	hnRNP A1, cAMP-GEFI, alphaAPPs, CED-6, AP4M1, GBR1, Calsyntenin-1, APPBP2, APP-C99, Pin1, SORL1, ITM2B, APP, DAB2, ZNF370, APLP1 precursor, RanBPM, Rac1, NGF, GSK3 beta, betaAPPs, ADAM9, Netrin-1, LDLRAD3, PLC-gamma 1, Adenylate cyclase, Tiam2, Casein kinase II, ZDHHC21, Amyloid beta 40, SFRS2 (SC-35), ACAT1, Amyloid beta, ADAM10, Fe65, SFK, c-Src, ADAM17, APP-C59 (AICD), ITM2C, LRP1, Amyloid beta 42, RAP6
**15**	Androgen receptor activation and downstream signaling in Prostate cancer	1.768E-09	E-cadherin, SLC45A3, TGF-beta 1, B-Raf, GRB2, FGFR2, JAK1, K-RAS, c-Cbl, GCR, NCOA3 (pCIP/SRC3), TMPRSS2, PKC-delta, IRS-1, EGFR, FGF1, Clusterin, c-Raf-1, FEN1, APP, p21, Bak, NCOA2 (GRIP1/TIF2), Elk-4, FGFR1, STAT3, ADAM9, PPAP, PTEN, N-Ras, Kallikrein 2, MEK2(MAP2K2), Bcl-XL, NCOA1 (SRC1), PAR1, c-FLIP, ERK2 (MAPK1), ADAM10, HNF3-alpha, c-Src, ADAM17, ERK1 (MAPK3), Prosaposin
**16**	IGF family signaling in colorectal cancer	2.459E-09	PI3K reg class IA (p85-alpha), GRB2, CDC25C, IRS-2, IRS-1, E2F1, ERK1/2, Clusterin, Beta-catenin, c-Raf-1, IKK (cat), IGF-2 receptor, Rad51, I-kB, PI3K cat class IA (p110-alpha), ZNF143, GSK3 beta, p70 S6 kinase1, PTEN, IKK-alpha, GSK3 alpha/beta, Bcl-XL, MNK2(GPRK7), MEK1/2, ERK2 (MAPK1), ERK1 (MAPK3), mTOR, FosB, C/EBPalpha
**17**	G-protein signaling_RhoA inhibition	3.213E-09	RhoA, Fbxl19, 14-3-3 epsilon, Cyclin B, cAMP-GEFI, RHO6, SUMO-1, p190RhoGAP, GEF720, TGF-beta, PKA-reg (cAMP-dependent), RhoGAP5, E2I, Myosin IXb, Alpha-catenin, Rac1, ACP1, NGF, PRKD1, ARHGEF2, NET1, 14-3-3 sigma, Casein kinase II, RHG7, CDC42, Fyn, Aurora-B, PRNP, ERK2 (MAPK1), RacGAP1, c-Src, ERK1 (MAPK3), PKA-cat (cAMP-dependent), SLK
**18**	DNA damage_ATM activation by DNA damage	3.375E-09	Casein kinase II, alpha chains, CDK5, Brca1, WWOX, BRAT1, Histone H3, EGFR, INTS3, PP2A regulatory, Rad50, Tip60, p18, MRE11, Pellino 1, MRG15, IHPK2, HDAC2, MRN complex, NK31, ATM, Casein kinase II, alpha chain (CSNK2A1), FOXO3A, Casein kinase II, RNF8, ASCIZ, TELO2, eIF3S6, HDAC1, CHFR, HSP90, Nibrin, NDR1 (STK38)
**19**	Neurophysiological process_Dynein-dynactin motor complex in axonal transport in neurons	3.387E-09	CDK5, Huntingtin, ERK1/2, DCTN1(p150Glued), Importin (karyopherin)-alpha, Sortilin, BPAG1, HDAC6, APP, JSAP1, Ubiquitin, NGF, Centractins, DYNLL, ORP1, Dynein 1, cytoplasmic, heavy chain, Tctex-1, Alpha-centractin, Kinesin heavy chain, DYNLT, HAP40, PRNP, Kinesin light chain, Dynein 1, cytoplasmic, intermediate chains, MAPRPE1(EB1), DYI2, SPTBN2

**20**	IGF-1 signaling in multiple myeloma	1.291E-08	RhoA, PI3K cat class IA, GRB2, IRS-2, IRS-1, ERK1/2, c-FLIP(Long), c-Raf-1, IKK (cat), Fibronectin, I-kB, PRKD1, FOXO4, p70 S6 kinase1, PI3K reg class IA, PTEN, IKK-alpha, FOXO3A, GSK3 alpha/beta, MEK1/2, CDC42, ERK2 (MAPK1), ERK1 (MAPK3), Bim, mTOR
**21**	Development_NOTCH signaling inhibition	1.436E-08	Casein kinase II, alpha chains, c-Cbl, CIR, FBXW7, MAML1, CDK8, J1ICD, LSD1, EGFL7, ILK, RBB2, NOTCH4 (ICD4), NLK, Tip60, Sirtuin1, GSK3 beta, FHL1 (SLIM1), HDAC2, NEURL1, BMPR1B, Elongin C, MMP-14, NOTCH4 receptor, N-CoR, Skp2/TrCP/FBXW, WDR48, USP12, HDAC1, SAP30, ARRDC1, RBBP8 (CtIP), DEC1 (Stra13), Jagged1, Fe65, c-Src, Nibrin, APP-C59 (AICD), CBX4
**22**	Chemotaxis_Lysophosphatidic acid signaling via GPCRs	1.503E-08	RhoA, E-cadherin, cPKC (conventional), PLC-eta 1, PKC-delta, Tcf(Lef), EGFR, G-protein alpha-i family, LARG, MKL2, ERK1/2, Beta-catenin, c-Raf-1, HDAC7, Caspase-9, p21, LIMK, TRIP6, PLD2, Rac1, IP3 receptor, E3KARP (NHERF2), MEK4(MAP2K4), PRKD1, PI3K reg class IA (p85), Bax, GSK3 beta, Caspase-7, p70 S6 kinase1, PKC, Vinculin, ATF-2, Caspase-3, PLC-delta 1, FasR(CD95), Bcl-XL, MEK1/2, Rho GTPase, CDC42, N-CoR, TAZ, c-Src, ADAM17, PLC-epsilon, mTOR, PREX1
**23**	Cell cycle_Sister chromatid cohesion	1.538E-08	TOP1, Separase, Histone H3, SMC1, DERPC, PDS5, Securin, PCNA, Mitotic cohesin complex, SMC3, ATM, DNA polymerase sigma, Rad21, Stromalins 1/2, DCC1
**24**	Transcription_Negative regulation of HIF1A function	2.887E-08	MCM3, Sirtuin3, MCM5, PRDX2, CITED2, COMMD1 (MURR1), FBXW7, ARD1, VCP, Sirtuin2, AML1 (RUNX1), Ubiquitin, Sirtuin1, GSK3 beta, MCM7, FHL1 (SLIM1), FHL2, PTEN, SSAT, LAMP2, Elongin C, OS-9, FIH-1, MCM2, HSP90, PRDX4, CHIP, HSP70, CITED4, SAT2
**25**	DNA damage_ATM-dependent double-strand break foci	3.681E-08	JMJD2A, UFD1, Brca1, Histone H3, BBAP, STARING, PIAS4, RNF168, KDM2A, BAF180, VCP, PIAS1, WHSC1, E2I, p53BP1, MRN complex, BAL, Mi-2 beta, ATM, RNF8, Mi-2 alpha, RING2, SMARCA5, Brca1/Bard1, Bcl-10, Bard1, Nibrin, RNF20, RAP80
**26**	Cell cycle_The metaphase checkpoint	3.688E-08	MIS12, HZwint-1, ZW10, CENP-A, CENP-H, SPBC25, Rod, DSN1, CENP-E, BUB1, CDCA1, CENP-F, MAD1 (mitotic checkpoint), Dynein 1, cytoplasmic, heavy chain, BUBR1, Aurora-B, HEC, HP1 alpha, NSL1, AF15q14
**27**	Oxidative stress_ROS signaling	3.868E-08	Casein kinase II, alpha chains, ACACA, SREBP1 (nuclear), Adrenomedullin, IRP1, SCD, PKA-cat alpha, ERK1/2, PKA-reg (cAMP-dependent), IKK (cat), Pin1, Glutaredoxin 1, p21, Bak, FASN, E2I, HSPA1A, PRKD1, Bax, Sirtuin1, GSK3 beta, p70 S6 kinase1, PKC, PTEN, HES1, FTL, IKK-alpha, HSP27, ATM, IKK-beta, DLC1 (Dynein LC8a), HDAC1, FIH-1, SAE2, SP1, Cyclin B1, NRF2, c-Src, ADAM17, mTOR
**28**	Ligand-independent activation of Androgen receptor in Prostate Cancer	5.532E-08	PI3K cat class IA, B-Raf, GRB2, FGFR2, K-RAS, NCOA3 (pCIP/SRC3), IRS-1, Tcf(Lef), EGFR, FGF1, Beta-catenin, PP2A regulatory, c-Raf-1, NCOA2 (GRIP1/TIF2), DDX5, STAT5B, FGFR1, STAT3, Tip60, GSK3 beta,

			PI3K reg class IA, N-Ras, MEK2(MAP2K2), Bcl-XL, NCOA1 (SRC1), HDAC1, ERK2 (MAPK1), ERK1 (MAPK3), Frizzled
**29**	Signal transduction_FGFR4 signaling	5.890E-08	PI3K cat class IA, GGPS1, GRB2, SR-BI, FGFR4, S100B, ERK1/2, FGF1, SC4MOL, RAS, c-Raf-1, PI3K cat class III (Vps34), MVK, DHCR7, IDI1, STAT1, I-kB, PI3K reg class IA (p85), ACLY, FGF18, Schwannomin (NF2), PKC, ERG1, LDLR, IKK-beta, PLC-gamma, Beclin 1, MEK1/2, INSIG1, SFK, SHIP2, DHC24, mTOR
**30**	Signal transduction_IGF-1 receptor signaling	7.023E-08	14-3-3 epsilon, PI3K cat class IA, Calcineurin A (catalytic), Brca1, SREBP1 (nuclear), GRB2, JAK1, IRS-2, SCD, IRS-1, ASK1 (MAP3K5), G-protein alpha-i family, ERK1/2, Clusterin, Beta-catenin, c-Raf-1, IKK (cat), FASN, SH2B, MEK4(MAP2K4), STAT3, I-kB, G-protein alpha-i1, PI3K reg class IA (p85), NF-AT4(NFATC3), GSK3 beta, p70 S6 kinase1, PCAF, PLC-gamma 1, FOXO3A, MMP-14, Bcl-XL, MNK2(GPRK7), MEK1/2, NRF2, p27KIP1, Bim, LRP1, mTOR
**31**	Apoptosis and survival_Cytoplasmic/mitochondrial transport of proapoptotic proteins Bid, Bmf and Bim	7.151E-08	HGK(MAP4K4), N-myristoyltransferase, DLC2 (Dynein LC8b), Apaf-1, ASK1 (MAP3K5), GLK(MAP4K3), DAXX, TRADD, Caspase-9, FLASH, MEK4(MAP2K4), Bax, Caspase-10, tBid, DLC1 (Dynein LC8a), FasR(CD95), GCKR(MAP4K5), Bim, Bid
**32**	Transcription_Epigenetic regulation of gene expression	7.344E-08	HDAC9, HAT1, Sirtuin3, Histone H3, PLU-1, UTX, DNMT3A, HDAC6, SMCX, LSD1, JMJD1A, Sirtuin2, RBB2, HBOA, Tip60, MLL1 (HRX), Sirtuin1, HDAC2, PCAF, HDAC8, DNMT1, HDAC1, MORF, JMJD3, G9a, SET7
**33**	Signal transduction_mTORC2 downstream signaling	8.222E-08	RhoA, Rictor, cPKC (conventional), ACACA, OSR1, SREBP1 (nuclear), SCD, PKC-delta, IRS-1, Beta-catenin, PKA-reg (cAMP-dependent), p21, FASN, FBX29, Rac1, GSK3 beta, PKC, PKC-alpha, Insulin receptor, FOXO3A, NEDD4L, GSK3 alpha/beta, CDC42, HXK4, p27KIP1, Bim, PKA-cat (cAMP-dependent), mTOR, PREX1
**34**	Role of ER stress in obesity and type 2 diabetes	9.097E-08	AdipoR2, PI3K cat class IA, C/EBP zeta, PI3K reg class IA (p85-alpha), SREBP1 (nuclear), eIF2AK3, IRS-2, PKR, SCD, IRS-1, FASN, SREBP1 (Golgi membrane), S1P, SREBP1 precursor, PI3K reg class IA (p85), HMGCS1, FDPS, BI-1, LDLR, Insulin receptor, FDFT1, HXK4, DnaJB9, PPAR-gamma, C/EBPalpha
**35**	Epithelial-to-mesenchymal transition (EMT) pathways in cancer cells	1.115E-07	E-cadherin, TGF-beta 1, PI3K cat class IA, GRB2, JAK1, EGFR, Beta-catenin, IKK (cat), TRADD, DDX5, ILK, STAT1, STAT3, I-kB, GSK3 beta, PI3K reg class IA, IKK-alpha, IKK-beta, PDGF receptor, c-Src, Axin, TAK1(MAP3K7), mTOR, PDGF-R-beta
**36**	DNA damage_Mismatch repair	1.386E-07	MutSalpha complex, Histone H3, MutLalpha complex, DNA-PK, PMS2, HDAC10, DNA ligase I, MSH6, WDHD1, PCNA, MRE11, MSH2, RPRD1B, HYPB, Casein kinase II, DNMT1, MLH1, Artemis

**37**	SCAP/SREBP Transcriptional Control of Cholesterol and FA Biosynthesis	1.612E-07	ACACA, GGPS1, SREBP1 (nuclear), SCD, FADS2, HMGCS2, LSS, FASN, SREBP1 (Golgi membrane), MVK, DHCR7, IDI1, SREBP1 precursor, ACLY, GSK3 beta, HMGCS1, FDPS, EBP, ERG1, ACSS1, FDFT1, INSIG1
**38**	Stellate cells activation and liver fibrosis	1.759E-07	COL1A1, Biglycan, TGF-beta 1, PI3K cat class IA, GRB2, ICAM1, Tcf(Lef), Beta-catenin, c-Raf-1, IKK (cat), TRADD, DAB2, SMAD2, Smoothened, I-kB, PI3K reg class IA (p85), GSK3 beta, MyD88, TRAF1, RIPK1, MEK2(MAP2K2), ACTA2, ERK2 (MAPK1), SP1, PDGF receptor, ERK1 (MAPK3), Frizzled, TGF-beta receptor type I, PDGF-R-beta
**39**	Cell cycle_Chromosome condensation in prometaphase	1.833E-07	TOP1, Cyclin B, Histone H3, CAP-G, CAP-G/G2, CAP-C, CAP-D2/D3, CAP-E, Condensin, CAP-H/H2, CNAP1, Aurora-B, TOP2, BRRN1
**40**	Apoptosis and survival_Regulation of apoptosis by mitochondrial proteins	2.253E-07	NIX, PLSCR3, Calcineurin A (catalytic), NOR1, Aif, SMCR7, PKC-delta, Fis1, Apaf-1, Cathepsin H, ERK1/2, MAP1, Pin1, PP2C, Caspase-9, Bak, Cathepsin S, MFF, OPA1, JSAP1, Cathepsin B, CDK2, Bax, OMA1, Caspase-10, SOD1, PINK1, ATF-2, SLC25A3, tBid, Beclin 1, Bcl-XL, MTCH2, HtrA2, VDAC 1, Mitofusin 2, Bim, Bid
**41**	E-cadherin signaling and its regulation in gastric cancer	2.418E-07	RhoA, E-cadherin, GRB2, FGFR2, EGFR, FGF1, Beta-catenin, Actin, Alpha-catenin, Ubiquitin, GSK3 beta, WNT, DNMT1, VAV-3, Plakoglobin, HGF receptor (Met), p120-catenin, c-Src, Frizzled
**42**	Signal transduction_PTEN pathway	2.637E-07	PI3K cat class IA, GRB2, c-Cbl, IRS-1, Tcf(Lef), EGFR, ERK1/2, Beta-catenin, c-Raf-1, Caspase-9, p21, ILK, PCNA, GSK3 beta, PI3K reg class IA, PTEN, Caspase-3, FOXO3A, MEK2(MAP2K2), MAGI-3, c-Src, mTOR
**43**	DNA damage_ATM/ATR regulation of G1/S checkpoint	5.150E-07	Brca1, hnRNP K, FBXW7, ERK1/2, PP2A regulatory, BTG2, p21, PER3, CDC27, CDK2, PCNA, p70 S6 kinases, MRN complex, PP2A structural, ATM, FOXO3A, MEK2(MAP2K2), Brca1/Bard1, p27KIP1, FBXW11, RFWD3
**44**	Tau dysregulation in Alzheimer disease	5.668E-07	CDK5, Calcineurin A (catalytic), SUMO-1, SET, ERK1/2, PHAP1 (pp32), PP2A regulatory, HDAC6, Caspase-9, Alpha-2A adrenergic receptor, APP, PPP2R5E, RanBPM, FKBP5, p70 S6 kinases, Sirtuin1, GSK3 beta, RBBP7 (RbAp46), CIP2A, Caspase-3, LCMT1, Fyn, OGT (GlcNAc transferase), Legumain, PRNP, PPME1, HSP90, CHIP, Fe65, APP-C59 (AICD), PKA-cat (cAMP-dependent), Amyloid beta 42
**45**	Main growth factor signaling cascades in multiple myeloma cells	6.172E-07	PI3K cat class IA, PI3K reg class IA (p85-alpha), GRB2, K-RAS, IRS-2, IRS-1, VEGFR-1, c-Raf-1, IKK (cat), STAT3, I-kB, PI3K reg class IA (p85), PI3K reg class IA, PTEN, IKK-alpha, N-Ras, MEK2(MAP2K2), GSK3 alpha/beta, ERK2 (MAPK1), ERK1 (MAPK3)

**46**	Autophagy_Autophagy	6.292E-07	APG16L1, C/EBP zeta, Huntingtin, VAMP8, eIF2AK3, GATE-16, GABARAP, Barkor, APG7, SNAP-29, Sec8, PI3K cat class III (Vps34), GABARAPL1, Tip60, Sirtuin1, GSK3 beta, UVRAG, RalB, WIP1, PKC-alpha, PINK1, ATG13, Beclin 1, SUR-8, NBR1, mTOR, FIP200
**47**	Translation_Regulation of EIF2 activity	7.257E-07	Casein kinase II, alpha chains, Casein kinase II, beta chain (Phosvitin), PI3K cat class IA, GRB2, eIF2AK3, c-Cbl, IRS-2, PKR, IRS-1, EGFR, ERK1/2, c-Raf-1, DYRK2, PI3K reg class IA, Insulin receptor, GSK3 alpha/beta, MEK1/2, Casein kinase I, eIF2AK1
**48**	Development_Negative regulation of WNT/Beta-catenin signaling at the receptor level	8.246E-07	Tsukushi, AP complex 2 medium (mu) chain, Tcf(Lef), FZD4, DAB2, Krm, LRP4, ZNRF3, WNT, Syndecans, Casein kinase II, GSK3 alpha/beta, Clathrin, SFRP, Amyloid beta, c-Src, Axin, GRB10, SNX27, LRP1, Frizzled
**49**	Mechanisms of resistance to EGFR inhibitors in lung cancer	8.246E-07	E-cadherin, PI3K cat class IA, GRB2, K-RAS, c-Cbl, IRS-1, EGFR, Beta-catenin, c-Raf-1, Fibronectin, PI3K reg class IA (p85), p70 S6 kinase1, PTEN, MEK2(MAP2K2), Claudin-4, ERK2 (MAPK1), HSP90, HGF receptor (Met), c-Src, ERK1 (MAPK3), mTOR
**50**	Immune response_IFN-gamma signaling via PI3K and NF-kB	9.568E-07	Casein kinase II, alpha chains, Rictor, PI3K cat class IA, PI3K reg class IA (p85-alpha), JAK1, PKR, PKC-delta, ERK1/2, Beta-catenin, IFNGR2, c-Raf-1, IRF1, SIN1, STAT1, I-kB, GSK3 beta, IKK-alpha, IKK-beta, MEK1/2, STAT5, Fyn, SP1, mTOR, PD-L1

Supplementary Table A5. List of the top 50 differentially expressed gene (DEG)-enriched signaling pathways within the Marburg virus (MARV)-infected human *in vitro* model. The listed DEG-enriched signaling pathways were associated with 682 DEGs within the GSE226148 dataset, generated by MetaCore, and sorted in descending order of log(*p* values).

#	Maps	p-value	Network Objects from Active Data
1	Immune response_IFN-alpha/beta signaling via JAK/STAT	1.925E-26	WNT7B, Axin2, Fibronectin, WNT, NRCAM, Axin, MITF, SOX9, Frizzled
2	COVID-19: Regulation of antiviral response by SARS-CoV-2	2.324E-16	CCL5, IRF1, MDA-5, RIG-I, HIF1A, TLR3, Fibronectin, CaMK IV, VCAM1, IFI56, STAT1, IP10, CSF1
3	Immune response_IFN-alpha/beta signaling via MAPKs	7.326E-11	ROR-alpha, Alpha-1B adrenergic receptor, Adrenomedullin, MGF, CITED2, Heme oxygenase 1, Oct-3/4, Thrombospondin 1, HIF1A, Carbonic anhydrase XII, CXCR4, Lysyl oxidase, GPI

**4**	Cigarette smoke-mediated regulation of NRF2-antioxidant pathway in airway epithelial cells	1.057E-10	DR4(TNFRSF10A), TRUNDD(TNFRSF10D), c-IAP1, c-IAP2, Caspase-7, XIAP, Apo-2L(TNFSF10), Osteoprotegerin
**5**	Attenuation of IFN type I signaling in melanoma cells	3.275E-08	GCL cat, Sequestosome 1(p62), Heme oxygenase 1, TXNRD1, PPARGC1 (PGC1-alpha), GSHR, NQO1, GCL reg, TXNIP (VDUP1), SLC7A11
**6**	Inhibition of Ephrin receptors in colorectal cancer	5.930E-08	NLK, BACH1, Oct-3/4, VHL, Tcf(Lef), WNT, KLF4, Axin, SOX9, TCF7L2 (TCF4), Frizzled, 14-3-3
**7**	Immune response_IFN-gamma signaling via MAPK	8.547E-08	CCL5, IRF1, IRF8, BATF2, Thrombospondin 1, CYP27B1, TLR4, STAT1, IP10
**8**	Immune response_Antiviral actions of Interferons	1.058E-07	IRF8, EPAS1, HIF1A, ID1, KLF4, TLR4, SLC7A11, STAT1, PD-L1, CSF1
**9**	Immune response_Antimicrobial actions of IFN-gamma	1.561E-07	DR4(TNFRSF10A), c-IAP1, c-IAP2, ISGF3, Caspase-7, XIAP, Apo-2L(TNFSF10)
**10**	Zika virus infection mechanism	2.475E-07	GCL cat, BACH1, Heme oxygenase 1, TXNRD1, NQO1, GCL reg, MAFG, ENC1, SLC7A11
**11**	Immune response_Induction of apoptosis and inhibition of proliferation mediated by IFN-gamma	3.829E-07	Oct-3/4, Tcf(Lef), WNT, TCF7L2 (TCF4), Frizzled, Neuropilin-1, PMP22
**12**	WNT signaling in proliferative-type melanoma cells	5.583E-07	CCL5, IRF1, IRF7, MyD88, MDA-5, RIG-I, HIF1A, TLR3, CXCL16, IP10, CSF1, ISG15
**13**	Glomerular injury in Lupus Nephritis	1.050E-06	MyD88, HSP70, Heme oxygenase 1, TXNRD1, NQO1, TLR4, MAFG
**14**	Transcription_HIF-1 targets	1.525E-06	DR4(TNFRSF10A), TRUNDD(TNFRSF10D), c-IAP1, c-IAP2, Caspase-7, XIAP, Apo-2L(TNFSF10)
**15**	Role of Apo-2L(TNFSF10) in Prostate Cancer cell apoptosis	2.548E-06	DR4(TNFRSF10A), WNT, VCAM1, IL-7, Frizzled, Apo-2L(TNFSF10), Osteoprotegerin
**16**	Oxidative stress_Role of Sirtuin1 and PGC1-alpha in activation of antioxidant defense system	4.082E-06	DAB2, VHL, Porf-2, Tcf(Lef), Prickle-1, Skp2/TrCP/FBXW, WNT, Axin, Frizzled, G-protein alpha-13
**17**	Development_Negative regulation of WNT/Beta-catenin signaling in the nucleus	4.524E-06	NLRC5, IRF1, PSMB9, TAP1 (PSF1), HLA-F, HLAC, HLAB, STAT1
**18**	Immune response_IFN-gamma in macrophages activation	6.449E-06	WNT7B, Tcf(Lef), Prickle-1, WNT, Axin, TCF7L2 (TCF4), Frizzled
**19**	Myeloid-derived suppressor cells and M2 macrophages in cancer	7.452E-06	IRF1, GCH1, Pyk2(FAK2), OAS1, OAS2, 2'-5'-oligoadenylate synthetase, STAT1, IP10
**20**	Role of IFN-beta in activation of T cell apoptosis in multiple sclerosis	1.168E-05	NLK, WIP1, Tcf(Lef), WNT, SOX4, SOX9, XIAP, TCF7L2 (TCF4), Frizzled
**21**	NRF2 regulation of oxidative stress response	1.245E-05	WNT7B, WNT, Osteopontin, CXCR4, Frizzled, Osteoprotegerin
**22**	Development_WNT/Beta-catenin signaling in organogenesis	1.474E-05	CCL5, IRF1, IRF9, TLR4, STAT1, IP10

23	COVID-19: immune dysregulation	1.534E-05	CCL5, PPARGC1 (PGC1-alpha), IL7RA, TIGIT, IFI56, CXCR4, MKP-1, IP10, ISG15
24	The role of KEAP1/NRF2 pathway in skin sensitization	1.843E-05	DR4(TNFRSF10A), c-IAP1, c-IAP2, Caspase-7, XIAP, XAF1, Apo-2L(TNFSF10)
25	Resistance of pancreatic cancer cells to death receptor signaling	2.285E-05	IRF1, PKR, TLR3, CYP1B1, HLAB, STAT1, PD-L1, IP10
26	Role of osteoblasts in bone lesions formation in multiple myeloma	3.434E-05	IRF1, IRF7, MyD88, PKR, TLR3, Caspase-7, TLR4, STAT1
27	Development_Negative regulation of WNT/Beta-catenin signaling in the cytoplasm	6.155E-05	Oct-3/4, KLF5, Tcf(Lef), WNT, KLF4, TCF7L2 (TCF4), Frizzled
28	Immune response_Induction of the antigen presentation machinery by IFN-gamma	7.956E-05	PTCH1, SUFU, WNT, TCF7L2 (TCF4), Frizzled, Smoothened
29	WNT signaling in hepatocellular carcinoma (HCC)	8.479E-05	PTCH1, FZD1, SUFU, WNT, Frizzled, Smoothened
30	Immune response_IFN-gamma actions on extracellular matrix and cell differentiation	9.123E-05	GCL cat, DNAJB11, Sequestosome 1(p62), GADD34, PPARGC1 (PGC1-alpha), NQO1, GRP78, C/EBP zeta
31	Development_Positive regulation of WNT/Beta-catenin signaling in the nucleus	9.295E-05	SLFN5, IRF7, IFI17, GBP1, ISG54, RSAD2, NMI, IFIT1, Apo-2L(TNFSF10), ISG15
32	Bone metastases in Prostate Cancer	1.037E-04	Axin2, FZD1, Oct-3/4, Tcf(Lef), WNT, Axin, FZD5, TCF7L2 (TCF4), Frizzled
33	Cigarette smoke-mediated attenuation of antibacterial and antivirus immune response	1.037E-04	CCL5, PKR, MDA-5, RIG-I, TLR3, STAT1
34	Immune response_Glucocorticoid receptor immunological signaling	1.042E-04	DAB2, WNT, Axin, TCF7L2 (TCF4), Frizzled
35	Inhibition of apoptosis in gastric cancer	1.171E-04	EPAS1, PERC, Thrombospondin 1, HIF1A, CD137 ligand(TNFSF9), TLR4, Apo-2L(TNFSF10), STAT1, IP10, CSF1
36	Immune response_IFN-gamma signaling via PI3K and NF-kB	1.188E-04	Tcf(Lef), Skp2/TrCP/FBXW, WNT, Axin, TCF7L2 (TCF4), Frizzled
37	Immune response_Role of PKR in stress-induced antiviral cell response	1.350E-04	Kalirin, Thrombospondin 1, Ephrin-B, Ephrin-B receptors, STAT1, Collagen IV
38	Development_WNT/Beta-catenin signaling in embryogenesis	1.367E-04	CITED2, Thrombospondin 1, ID1, VDR, CYP27B1, CYP24A1, TCF7L2 (TCF4)
39	SHH signaling in colorectal cancer	1.537E-04	CCL5, IRF1, MyD88, C/EBP, TLR4, CXCR4, STAT1, ENA-78
40	Development_Embryonal epaxial myogenesis	1.537E-04	WNT7B, Axin2, Fibronectin, WNT, NRCAM, Axin, MITF, SOX9, Frizzled
41	Apoptosis and survival_Endoplasmic reticulum stress response	1.729E-04	CCL5, IRF1, MDA-5, RIG-I, HIF1A, TLR3, Fibronectin, CaMK IV, VCAM1, IFI56, STAT1, IP10, CSF1

**42**	Immune response_IFN-alpha/beta signaling via PI3K and NF-kB pathways	2.174E-04	ROR-alpha, Alpha-1B adrenergic receptor, Adrenomedullin, MGF, CITED2, Heme oxygenase 1, Oct-3/4, Thrombospondin 1, HIF1A, Carbonic anhydrase XII, CXCR4, Lysyl oxidase, GPI
**43**	WNT signaling in the progression of lung cancer	2.190E-04	DR4(TNFRSF10A), TRUNDD(TNFRSF10D), c-IAP1, c-IAP2, Caspase-7, XIAP, Apo-2L(TNFSF10), Osteoprotegerin
**44**	Mpox: monkeypox virus immune evasion	2.622E-04	GCL cat, Sequestosome 1(p62), Heme oxygenase 1, TXNRD1, PPARGC1 (PGC1-alpha), GSHR, NQO1, GCL reg, TXNIP (VDUP1), SLC7A11
**45**	Stem cells_Aberrant Wnt signaling in medulloblastoma stem cells	3.130E-04	NLK, BACH1, Oct-3/4, VHL, Tcf(Lef), WNT, KLF4, Axin, SOX9, TCF7L2 (TCF4), Frizzled, 14-3-3
**46**	Macrophage and dendritic cell phenotype shift in cancer	3.608E-04	CCL5, IRF1, IRF8, BATF2, Thrombospondin 1, CYP27B1, TLR4, STAT1, IP10
**47**	WNT signaling in gastric cancer	3.628E-04	IRF8, EPAS1, HIF1A, ID1, KLF4, TLR4, SLC7A11, STAT1, PD-L1, CSF1
**48**	CHDI_Correlations from Discovery data_Causal network (positive)	3.628E-04	DR4(TNFRSF10A), c-IAP1, c-IAP2, ISGF3, Caspase-7, XIAP, Apo-2L(TNFSF10)
**49**	Inhibition of Calcitriol/ VDR signaling in colorectal cancer	4.083E-04	GCL cat, BACH1, Heme oxygenase 1, TXNRD1, NQO1, GCL reg, MAFG, ENC1, SLC7A11
**50**	Inflammatory mechanisms of pancreatic cancerogenesis	4.219E-04	Oct-3/4, Tcf(Lef), WNT, TCF7L2 (TCF4), Frizzled, Neuropilin-1, PMP22

Supplementary Table A6: List of potential compounds identified by a connectivity map (CMap) analysis based on 90 common differentially expressed genes (DEGs) in the Marburg virus (MARV)-infected group compared to the corresponding mock-infected group of both models. Potential compounds with fdr_q_nlog10 (negative log10-transformed false discovery rate (FDR) q-values) of ≥ 2.0 were sorted in ascending order of positive normalized connectivity scores.

Rank	Compounds	Cell name	Dose	Time	Mechanism of Action	FDR_q_nlog10	Normalized connectivity score (norm_cs)
1	AV-412	HA1E	2.22 uM	24 h	Receptor tyrosine protein kinase inhibitor	15.65	2.27
2	SB-218078	SKB	10 uM	24 h	CHK inhibitor	3.27	2.23
3	DPI-201106	HA1E	10 uM	24 h	Sodium channel activator	3.05	2.21
4	suloctidil	SKB	10 uM	24 h	Adrenergic receptor antagonist	2.88	2.18
5	BIX-01294	SKL	10 uM	24 h	DNA inhibitor|Histone lysine methyltransferase inhibitor	2.85	2.18
6	UNC-0638	SKL	10 uM	24 h	HMTase inhibitor	2.69	2.13

**7**	simeprevir	HA1E	10 uM	24 h	HCV inhibitor	2.34	1.96
**8**	GSK-461364	SKL	10 uM	24 h	PLK inhibitor	2.33	1.95
**9**	alectinib	HA1E	10 uM	24 h	ALK inhibitor	2.31	1.94
**10**	BRD-K61033289	MCF7	10 uM	48 h	PPAR receptor agonist	2.30	1.93
**11**	NVP-AUY922	U2OS	10 uM	6 h	HSP inhibitor	2.24	1.88
**12**	fenretinide	SKB	10 uM	24 h	Apoptosis stimulant|Retinoid receptor agonist	2.23	1.87
**13**	SR-33805	HA1E	10 uM	24 h	Calcium channel blocker	2.22	1.86
**14**	radicicol	MCF7	1.11 uM	6 h	HSP inhibitor	2.22	1.86
**15**	ispinesib	SKB	10 uM	24 h	Kinesin inhibitor	2.21	1.85
**16**	clofoctol	HA1E	10 uM	24 h	Bacterial protein synthesis inhibitor	2.21	1.85
**17**	ZK-164015	SKB	10 uM	24 h	Estrogen receptor antagonist	2.19	1.83
**18**	CA-074-Me	VCAP	10 uM	24 h	Cathepsin inhibitor	2.19	1.83
**19**	BRD-K49657628	HT29	10 uM	6 h	EGFR inhibitor|Tyrosine kinase inhibitor	2.17	1.82
**20**	thiostrepton	HT29	10 uM	6 h	Protein synthesis inhibitor	2.17	1.81
**21**	chelerythrine	HT29	10 uM	24 h	Protein kinase inhibitor	2.17	1.81
**22**	adapalene	THP1	2.22 uM	24 h	Retinoid receptor agonist	2.16	1.8
**23**	penfluridol	YAPC	10 uM	24 h	Calcium channel blocker	2.15	1.8
**24**	CA-074-Me	HT29	10 uM	24 h	Cathepsin inhibitor	2.15	1.79
**25**	clocortolone-pivalate	MCF7	4 uM	48 h	Steroid	2.14	1.78
**26**	BRD-K68552125	HEK293	0.12 uM	24 h	PKC activator	2.14	1.78
**27**	favipiravir	HELA	0.25 uM	24 h	RNA polymerase inhibitor	2.14	1.78
**28**	dexniguldipine	P1A82	10 uM	24 h	Calmodulin inhibitor	2.13	1.77
**29**	lylamine	HA1E	10 uM	24 h	Cannabinoid receptor agonist	2.13	1.77
**30**	AV-412	HT29	10 uM	24 h	Receptor tyrosine protein kinase inhibitor	2.12	1.76
**31**	xanthohumol	SKB	10 uM	24 h	ATPase inhibitor	2.12	1.76
**32**	SA-792709	SKB	10 uM	24 h	Retinoid receptor agonist	2.12	1.76
**33**	ionomycin	NPC	10 uM	24 h	Calcium channel blocker	2.11	1.74
**34**	amlodipine	MCF7	10 uM	48 h	Calcium channel blocker	2.10	1.74
**35**	BS-181	SKL	10 uM	24 h	CDK inhibitor	2.10	1.73
**36**	paroxetine	U2OS	10 uM	24 h	Selective serotonin reuptake inhibitor (SSRI)	2.09	1.73
**37**	alectinib	CD34	10 uM	24 h	ALK inhibitor	2.09	1.72
**38**	BRD-K28120222	HCC515	10 uM	6 h	NFKB inhibitor	2.09	1.72
**39**	fenretinide	HA1E	10 uM	24 h	Apoptosis stimulant|Retinoid receptor agonist	2.09	1.72
**40**	BRD-K68552125	HA1E	0.12 uM	24 h	PKC activator	2.09	1.72

**41**	ixazomib	HCC515	10 uM	6 h	Proteasome inhibitor	2.09	1.72
**42**	AG-592	HA1E	10 uM	24 h	Tyrosine kinase inhibitor	2.09	1.72
**43**	CA-074-Me	MCF7	10 uM	24 h	Cathepsin inhibitor	2.08	1.71
**44**	parthenolide	HT29	10 uM	6 h	NFKB inhibitor	2.08	1.71
**45**	NSC-663284	HT29	10 uM	6 h	CDC inhibitor	2.08	1.71
**46**	obatoclax	HA1E	10 uM	6 h	BCL inhibitor	2.08	1.7
**47**	CD-1530	P1A82	10 uM	24 h	Retinoid receptor agonist	2.08	1.7
**48**	pifithrin-mu	HCC515	10 uM	6 h	p53 inhibitor	2.07	1.7
**49**	AG-592	HT29	10 uM	6 h	Tyrosine kinase inhibitor	2.07	1.7
**50**	bindarit	HELA	0.08 uM	24 h	NFKB inhibitor	2.07	1.7
**51**	eptifibatide	HELA	2.22 uM	24 h	Platelet aggregation inhibitor	2.07	1.69
**52**	CGK-733	SKB	10 uM	24 h	ATM kinase inhibitor|ATR kinase inhibitor	2.07	1.69
**53**	suloctidil	HT29	10 uM	6 h	Adrenergic receptor antagonist	2.07	1.69
**54**	lylamine	U2OS	10 uM	6 h	Cannabinoid receptor agonist	2.07	1.69
**55**	spiperone	U2OS	10 uM	6 h	Dopamine receptor antagonist	2.06	1.69
**56**	BI-78D3	MCF7	10 uM	6 h	JNK inhibitor	2.06	1.68
**57**	devazepide	HCC515	10 uM	24 h	CCK receptor antagonist	2.06	1.68
**58**	BRD-K74305673	HA1E	10 uM	6 h	IKK inhibitor|NFKB inhibitor	2.06	1.68
**59**	tetrindole	HA1E	10 uM	24 h	Monoamine oxidase inhibitor	2.06	1.68
**60**	parthenolide	VCAP	10 uM	6 h	NFKB inhibitor	2.06	1.68
**61**	COL-3	HT29	10 uM	24 h	Matrix metalloprotease inhibitor	2.06	1.68
**62**	tyrphostin-47	HT29	10 uM	6 h	EGFR inhibitor	2.06	1.67
**63**	WAY-170523	HT29	10 uM	6 h	Metalloproteinase inhibitor	2.05	1.67
**64**	bosutinib	U2OS	10 uM	48 h	Src inhibitor|Abl inhibitor|Bcr-Abl inhibitor	2.05	1.67
**65**	HU-211	SKB	10 uM	24 h	Glutamate receptor antagonist	2.05	1.67
**66**	curcumin	U2OS	10 uM	48 h	Cyclooxygenase inhibitor|Histone acetyltransferase inhibitor|Lipoxygenase inhibitor|NFKB inhibitor	2.05	1.67
**67**	BAY-11-7082	MCF7	10 uM	6 h	NFKB inhibitor	2.05	1.67
**68**	econazole	HELA	10 uM	24 h	Lanosterol demethylase inhibitor|Bacterial cell wall synthesis inhibitor|Sterol demethylase inhibitor	2.05	1.67
**69**	amlodipine	U2OS	10 uM	6 h	Calcium channel blocker	2.05	1.66
**70**	BCI-hydrochloride	HT29	10 uM	6 h	Protein phosphatase inhibitor	2.05	1.66
**71**	thiomersal	HELA	2.22 uM	24 h	Antibiotic	2.05	1.66
**72**	BRD-K07303502	MCF7	80 uM	6 h	Cytosolic phospholipase inhibitor	2.04	1.65

**73**	SR-57227A	NPC	10 uM	24 h	Serotonin receptor agonist	2.04	1.65
**74**	STA-5326	HEK293	2.22 uM	24 h	Interleukin inhibitor	2.04	1.65
**75**	LY-2811376	HELA	0.01 uM	24 h	Beta secretase inhibitor	2.04	1.65
**76**	KIN-001-220	THP1	10 uM	24 h	Aurora kinase inhibitor	2.04	1.65
**77**	AG-957	MCF7	10 uM	6 h	Protein tyrosine kinase inhibitor	2.04	1.65
**78**	parthenolide	SKB	10 uM	24 h	NFKB inhibitor	2.04	1.65
**79**	pifithrin-mu	AGS	10 uM	6 h	p53 inhibitor	2.04	1.65
**80**	ixazomib	HA1E	0.125 uM	24 h	Proteasome inhibitor	2.03	1.64
**81**	CL-218872	SKB	10 uM	24 h	GABA receptor agonist	2.03	1.64
**82**	CAY-10594	HT29	20 uM	24 h	Phospholipase inhibitor	2.03	1.64
**83**	BRD-K18722736	NEU	10 uM	24 h	Opioid receptor antagonist	2.03	1.64
**84**	manumycin-a	HCC515	10 uM	6 h	Farnesyltransferase inhibitor|NFKB inhibitor	2.03	1.63
**85**	protriptyline	HA1E	10 uM	24 h	Tricyclic antidepressant	2.03	1.63
**86**	quizartinib	CD34	10 uM	24 h	FLT3 inhibitor	2.02	1.63
**87**	BRD-K07303502	HT29	80 uM	6 h	Cytosolic phospholipase inhibitor	2.02	1.63
**88**	ixazomib	HELA	2.22 uM	24 h	Proteasome inhibitor	2.02	1.63
**89**	BRD-K04546108	HCC515	10 uM	6 h	JAK inhibitor	2.02	1.62
**90**	exemestane	MCF7	10 uM	48 h	Aromatase inhibitor	2.02	1.62
**91**	phenytoin	HA1E	0.08 uM	24 h	Hydantoin antiepileptic	2.02	1.62
**92**	tetrindole	U2OS	10 uM	6 h	Monoamine oxidase inhibitor	2.02	1.62
**93**	7b-cis	AGS	10 uM	6 h	Exportin antagonist	2.02	1.62
**94**	sotrastaurin	OCILY3	10 uM	24 h	PKC inhibitor	2.01	1.61
**95**	BCL2-inhibitor	A375	10 uM	6 h	BCL inhibitor	2.01	1.61
**96**	BRD-U08759356	MCF7	10 uM	6 h	EGFR inhibitor	2.01	1.61
**97**	cyproheptadine	HT29	10 uM	6 h	Histamine receptor antagonist	2.01	1.61
**98**	CD-437	HCC515	12 uM	6 h	Retinoid receptor agonist	2.01	1.61
**99**	BRD-K25079130	HT29	10 uM	24 h	Nucleophosmin inhibitor	2.01	1.61
**100**	thapsigargin	MCF7	10 uM	24 h	ATPase inhibitor	2.01	1.61
**101**	brivudine	HELA	0.03 uM	24 h	DNA inhibitor	2.01	1.61
**102**	fluoxetine	U2OS	10 uM	6 h	Selective serotonin reuptake inhibitor (SSRI)	2.01	1.61
**103**	ASC-J9	HA1E	10 uM	24 h	Androgen receptor agonist	2.01	1.6
**104**	fidaxomicin	HELA	10 uM	24 h	RNA polymerase inhibitor	2.01	1.6
**105**	sofalcone	HA1E	10 uM	24 h	Mucus protecting agent	2.00	1.6

**106**	miconazole	HELA	2.22 uM	24 h	Bacterial cell wall synthesis inhibitor	2.00	1.6
**107**	phenethyl-isothiocyanate	HA1E	10 uM	6 h	Carcinogen	2.00	1.6
**108**	azacitidine	U2OS	10 uM	24 h	DNA methyltransferase inhibitor	2.00	1.6
**109**	BRD-A63236097	PC3	10 uM	24 h	Tumor necrosis factor production inhibitor	2.00	1.59
**110**	indirubin	MCF7	10 uM	24 h	CDK inhibitor|GSK inhibitor	2.00	1.59
**111**	arvanil	HA1E	10 uM	24 h	TRPV agonist	2.00	1.59
**112**	etravirine	A375	10 uM	24 h	Non-nucleoside reverse transcriptase inhibitor	2.00	1.59
**113**	navitoclax	U2OS	10 uM	48 h	BCL inhibitor	2.00	1.59
**114**	PD-160170	MCF7	10 uM	24 h	Neuropeptide receptor antagonist	2.00	1.59
**115**	pifithrin-mu	HT29	10 uM	6 h	p53 inhibitor	2.00	1.59
**116**	capsazepine	HA1E	10 uM	6 h	TRPV agonist	2.00	1.59
**117**	exemestane	MCF7	10 uM	24 h	Aromatase inhibitor	2.00	1.59

Supplementary Table A7: List of potential compounds identified by a connectivity map (CMap) analysis based on 90 common differentially expressed genes (DEGs) in the Marburg virus (MARV)-infected group compared to the corresponding mock-infected group of both models. Potential compounds with fdr_q_nlog10 (negative log10-transformed false discovery rate (FDR) q-values) of ≥ 2.0 were sorted in ascending order of negative normalized connectivity scores.

Rank	Compounds	Cell name	Dose	Time	Mechanism of Action	FDR_q_nlog10	Normalized connectivity score (norm_cs)
**1**	enalapril	MCF7	0.125 uM	24 h	ACE inhibitor	15.65	-1.88
**2**	GR-127935	U2OS	10 uM	6 h	Serotonin receptor antagonist	15.65	-1.84
**3**	KU-0063794	H1299	10 uM	6 h	MTOR inhibitor	15.65	-1.73
**4**	BRD-K14329163	NCIH2073	10 uM	6 h	Calcium channel activator	15.65	-1.71
**5**	westcort	A549	10 uM	6 h	Glucocorticoid receptor agonist	15.65	-1.67
**6**	KU-0063794	VCAP	10 uM	6 h	MTOR inhibitor	15.65	-1.66
**7**	clinofibrate	MCF7	2.22 uM	24 h	Lipase inhibitor	15.65	-1.63
**8**	AZ-628	HCC515	10 uM	6 h	RAF inhibitor	15.65	-1.63
**9**	carbenoxolone	A549	10 uM	6 h	11-beta-HSD1 inhibitor	15.65	-1.62

**10**	trimetazidine	HELA	0.01 uM	24 h	3-ketoacyl CoA thiolase inhibitor	15.65	-1.62
**11**	zolpidem	A549	10 uM	6 h	Benzodiazepine receptor agonist	15.65	-1.62
**12**	orlistat	MCF7	0.74 uM	24 h	Lipase inhibitor	15.65	-1.61
**13**	K-858	MCF7	10 uM	24 h	Kinesin inhibitor	15.65	-1.61
**14**	PI-103	HEPG2	12 uM	6 h	PI3K inhibitor|MTOR inhibitor	15.65	-1.61
**15**	PP-2	HT29	10 uM	6 h	Src inhibitor	15.65	-1.61
**16**	letermovir	PC3	10 uM	24 h	CMV terminase inhibitor	15.65	-1.6
**17**	BRD-K95992530	NEU	10 uM	24 h	Integrin inhibitor	15.65	-1.6
**18**	ebselen	A375	0.74 uM	24 h	Cyclooxygenase inhibitor|Glutathione peroxidase agonist|H+/K+-ATPase inhibitor|Nitric oxide synthase inhibitor	15.65	-1.6
**19**	lucitanib	HELA	10 uM	24 h	FGFR inhibitor|VEGFR inhibitor	15.65	-1.6
**20**	ranitidine	SKB	10 uM	24 h	Histamine receptor antagonist	15.65	-1.59
**21**	IOX2	CD34	10 uM	24 h	HIF Inhibitor	15.65	-1.59
**22**	selamectin	MCF7	10 uM	24 h	Nematocide	15.65	-1.59
**23**	MEK-162	MDAMB231	2.22 uM	24 h	MEK inhibitor	15.65	-1.58
**24**	equilin	U2OS	0.12 uM	6 h	Estrogen receptor agonist	15.35	-1.57
**25**	efaproxiral	A549	1.11 uM	24 h	Haemoglobin oxygen release stimulant	15.35	-1.57
**26**	BMS-536924	NCIH596	12 uM	6 h	IGF-1 inhibitor	15.35	-1.57
**27**	lomeguatrib	A549	0.04 uM	24 h	O6-alkylguanine-DNA alkyltransferase inhibitor	15.35	-1.56
**28**	clomethiazole	HT29	2.22 uM	24 h	GABA receptor antagonist|GABA receptor modulator	15.35	-1.56
**29**	isoconazole	MCF10A	10 uM	24 h	Fungal lanosterol demethylase inhibitor	15.35	-1.56
**30**	pidotimod	HA1E	3.33 uM	24 h	Interferon inducer|Interleukin receptor agonist	15.35	-1.55
**31**	linsitinib	HELA	0.25 uM	24 h	IGF-1 inhibitor	15.35	-1.55
**32**	SNX-2112	HT29	0.37 uM	24 h	HSP inhibitor	15.35	-1.55
**33**	mericitabine	A549	10 uM	24 h	HCV inhibitor	15.35	-1.55
**34**	pibenzimol	HT29	10 uM	24 h	DNA inhibitor	15.35	-1.55
**35**	wiskostatin	HEPG2	10 uM	6 h	Neural Wiskott-Aldrich syndrome protein inhibitor	15.35	-1.54
**36**	lenalidomide	NCIH2073	200 uM	6 h	Carcinogen	15.35	-1.54

**37**	levamisole	JURKAT	0.01 uM	24 h	Acetylcholine receptor agonist	15.35	-1.54
**38**	BRD-K23875128	HA1E	10 uM	6 h	Rho associated kinase inhibitor	15.35	-1.54
**39**	PD-0325901	A375	10 uM	6 h	MEK inhibitor|MAP kinase inhibitor|Protein kinase inhibitor	15.35	-1.54
**40**	hydrochlorothiazide	HEPG2	20 uM	24 h	Thiazide diuretic	15.35	-1.54
**41**	vemurafenib	HT29	10 uM	6 h	RAF inhibitor	15.35	-1.53
**42**	piribedil	MDAMB231	0.25 uM	24 h	Dopamine receptor agonist	15.35	-1.53
**43**	aniracetam	YAPC	0.37 uM	24 h	Glutamate receptor agonist	15.35	-1.53
**44**	AZD-8055	NPC	0.3 uM	24 h	MTOR inhibitor	15.35	-1.53
**45**	dantrolene	HELA	0.01 uM	24 h	Calcium channel blocker	15.35	-1.53
**46**	WZ-4002	HEPG2	10 uM	6 h	EGFR inhibitor	15.35	-1.53
**47**	AS-605240	AGS	10 uM	6 h	PI3K inhibitor	15.35	-1.53
**48**	methimazole	HA1E	0.01 uM	24 h	Antithyroid agent	15.35	-1.53
**49**	AZD-4547	ASC	10 uM	24 h	FGFR inhibitor	15.35	-1.53
**50**	reboxetine	PC3	0.25 uM	24 h	Adrenergic receptor antagonist	15.35	-1.52
**51**	biperiden	HELA	3.33 uM	24 h	Acetylcholine receptor antagonist	15.35	-1.52
**52**	TW-37	HA1E	0.37 uM	24 h	BCL inhibitor	15.35	-1.52
**53**	fasudil	THP1	10 uM	24 h	Rho associated kinase inhibitor	15.35	-1.52
**54**	U-0126	HEPG2	3.33 uM	6 h	MEK inhibitor	15.35	-1.52
**55**	T-0156	NPC	10 uM	24 h	Phosphodiesterase inhibitor	15.35	-1.52
**56**	ciprofloxacin	HPTEC	320 uM	24 h	Bacterial DNA Gyrase inhibitor	15.35	-1.52
**57**	TAK-733	MDAMB231	0.08 uM	24 h	MEK inhibitor	15.35	-1.51
**58**	LY-2228820	P1A82	10 uM	24 h	P38 MAPK inhibitor	15.35	-1.51
**59**	ML-130	HA1E	10 uM	6 h	NOD1 inhibitor	15.35	-1.51
**60**	AMZ-30	MCF7	10 uM	6 h	Protein phosphatase inhibitor	15.35	-1.51
**61**	nefopam	A375	10 uM	24 h	Cyclooxygenase inhibitor	15.35	-1.51
**62**	AZ-628	SKMEL5	0.66 uM	24 h	RAF inhibitor	15.35	-1.51
**63**	selumetinib	SW480	80 uM	6 h	MEK inhibitor	15.35	-1.5
**64**	valdecoxib	U2OS	10 uM	6 h	Cyclooxygenase inhibitor	15.35	-1.5
**65**	everolimus	MCF7	0.01 uM	24 h	MTOR inhibitor	15.35	-1.5

**66**	I-BET-151	VCAP	10 uM	24 h	Bromodomain inhibitor	15.35	-1.5
**67**	fraxetin	U2OS	10 uM	6 h	Antioxidant	15.35	-1.5
**68**	NVP-BEZ235	PHH	10 uM	24 h	MTOR inhibitor|PI3K inhibitor	15.35	-1.5
**69**	mexiletine	A549	10 uM	6 h	Sodium channel inhibitor	15.35	-1.5
**70**	fluprostenol	HCC515	10 uM	6 h	Prostanoid receptor agonist	15.35	-1.5
**71**	urapidil	HEK293	0.04 uM	24 h	Adrenergic receptor antagonist	15.35	-1.49
**72**	verapamil	HEPG2	10 uM	6 h	Calcium channel blocker	15.35	-1.49
**73**	lapatinib	HPTEC	100 uM	6 h	EGFR inhibitor|ERBB2 inhibitor	15.35	-1.49
**74**	doxylamine	SKB	10 uM	24 h	Histamine receptor antagonist	15.35	-1.49
**75**	cutamesine	YAPC	0.04 uM	24 h	Sigma receptor agonist	15.35	-1.49
**76**	tetramethylsilane	HT29	10 uM	6 h	Internal standard for NMR spectroscopy	15.35	-1.49
**77**	L-690330	NCIH596	10 uM	6 h	Inositol monophosphatase inhibitor	15.35	-1.49
**78**	timonacic	HT29	10 uM	24 h	Antioxidant	15.35	-1.49
**79**	BRD-K23875128	NCIH2073	10 uM	6 h	Rho associated kinase inhibitor	15.35	-1.49
**80**	senicapoc	PC3	1.11 uM	24 h	Potassium channel blocker	15.35	-1.49
**81**	AZ-628	A549	10 uM	6 h	RAF inhibitor	15.35	-1.49
**82**	PD-98059	A375	10 uM	24 h	MEK inhibitor|MAP kinase inhibitor	15.35	-1.49
**83**	fumonisin-b1	HT29	150 uM	24 h	Sphingosine kinase inhibitor	15.35	-1.49
**84**	atomoxetine	MCF7	0.125 uM	24 h	Norepinephrine inhibitor	15.35	-1.49
**85**	pseudopelletierine	VCAP	10 uM	24 h	Anthelmintic	15.35	-1.49
**86**	SEW-2871	HEPG2	10 uM	6 h	Lysophospholipid receptor agonist	15.35	-1.48
**87**	naphazoline	HUH7	15 uM	72 h	Adrenergic receptor agonist	15.35	-1.48
**88**	GDC-0941	A375	10 uM	6 h	PI3K inhibitor	15.35	-1.48
**89**	AS-703026	A375	2.22 uM	24 h	MEK inhibitor	15.35	-1.48
**90**	CCT-031374	U937	10 uM	6 h	WNT inhibitor	15.35	-1.48
**91**	ketotifen	MCF7	10 uM	24 h	Histamine receptor agonist|Leukotriene receptor antagonist|Phosphodiesterase inhibitor	4.08	-1.48
**92**	aliskiren	A375	3.33 uM	24 h	Antihypertensive|Peptidase inhibitor|Protease inhibitor|Renin inhibitor	3.15	-1.48
**93**	KBG	HEK293	0.37 uM	24 h	Neprilysin inhibitor	2.62	-1.48

**94**	BIIB-021	YAPC	0.08 uM	24 h	HSP inhibitor	2.24	-1.47
**95**	mannitol-D	MCF7	0.04 uM	24 h	Diuretic	2.17	-1.47
**96**	PD-0325901	HA1E	10 uM	6 h	MEK inhibitor|MAP kinase inhibitor|Protein kinase inhibitor	2.07	-1.47
**97**	AZD-7545	U937	20 uM	6 h	Pyruvate dehydrogenase kinase inhibitor	2.03	-1.47
**98**	fostamatinib	HA1E	2.22 uM	24 h	Syk inhibitor	2.03	-1.47
